# Immunomodulatory effects of dietary methionine supplementation in rainbow trout (*Oncorhynchus mykiss*) juveniles: insights following vaccination and infection response against *Yersinia ruckeri*

**DOI:** 10.3389/fimmu.2025.1706922

**Published:** 2025-11-03

**Authors:** Inês Carvalho, Felipe Bolgenhagen Schoninger, André Cunha, Diogo Peixoto, Francisca Brito, Luara Simões, Mariana Vaz, Allan Stensballe, Inês Ferreira, Paulo Santos, Carolina Tafalla, Marina Machado, Benjamín Costas

**Affiliations:** ^1^ Centro Interdisciplinar de Investigação Marinha e Ambiental (CIIMAR), Matosinhos, Portugal; ^2^ Instituto de Ciências Biomeédicas Abel Salazar (ICBAS), Universidade do Porto, Porto, Portugal; ^3^ Faculdade de Ciências da Universidade do Porto (FCUP), Porto, Portugal; ^4^ GreenCoLab, Associação Oceano Verde, Universidade do Algarve, Faro, Portugal; ^5^ Faculty of Mathematics and Natural Sciences, University of Bergen, Bergen, Norway; ^6^ Department of Health Science and Technology, University of Aalborg, Aalborg, Denmark; ^7^ Fish Immunology and Pathology Group, Biotechnology Department, National Institute for Agricultural and Food Research and Technology (INIA), Spanish National Research Council (CSIC), Madrid, Spain

**Keywords:** functional ingredients, amino acids, fish immunity, enteric redmouth disease, immunization

## Abstract

Methionine, an essential amino acid, participates in various pathways with implications for the immune system. Recent evidence suggests that it may support both innate and adaptive immune mechanisms. In the present study, it was hypothesized that dietary methionine supplementation prior to vaccination may be a promising strategy to improve vaccine efficacy. Hence, the current research aimed to evaluate the effects of dietary methionine supplementation on the immune status of rainbow trout (*Oncorhynchus mykiss*) juveniles, its role in modulating immune responses, as well as its potential synergistic effects with a commercial vaccine. To this end, fish were fed either a control diet (CTRL), meeting the methionine requirements of the species, or a methionine-supplemented diet (MET). After 4 weeks, half of the fish within each dietary group were either dip-vaccinated against *Yersinia ruckeri* (vaccinated) or left unvaccinated (naïve). Twenty-one days post-vaccination, during which fish continued on their respective dietary treatment, fish were intraperitoneally challenged with *Y. ruckeri* or injected with HBSS to serve as a control. Bacterial load in gills, posterior gut and spleen tissues, hematological parameters, differential cell counts, hepatic metabolites and antioxidant defenses, gene expression in the head-kidney and liver tissues, and plasma proteomic profiles were assessed following feeding trial and immunization period, and at early time points post-infection. Mortality was also monitored. Naïve fish exhibited a higher prevalence of *Y. ruckeri*, along with increased expression of pro-inflammatory and innate immune genes compared to their vaccinated counterparts. In contrast, vaccinated fish appeared to resolve the infection more rapidly, possibly through an early and heightened production of reactive oxygen species. In naïve fish, methionine supplementation appeared to impair antioxidant defenses and prolong immune activation, potentially contributing to the higher bacterial burden and reduced survival observed in this group. Differences between the two vaccinated groups were subtle, with no mortality recorded in either. However, proteomic analyses at 24 h post-infection revealed distinct responses, with MET-fed vaccinated fish exhibiting an increase in hemostasis-related proteins, while CTRL-fed vaccinated fish showed a response more akin to pre-infection groups. Methionine supplementation in combination with vaccination appeared to promote slightly faster pathogen clearance.

## 1 Introduction

Despite significant advances in aquaculture practices, disease outbreaks continue to pose major challenges, leading to substantial economic losses and threatening the sustainability of the industry (1). Vaccination has been a cornerstone of disease prevention (2), yet many vaccines used in fish production are still weakly immunogenic on their own (3–5). Given that formulated diets are the primary source of nutrients in intensive aquaculture, the enrichment of feed formulations with functional ingredients offers a promising approach not only to improve immune function, disease resistance (6, 7), and stress resilience (8), but also to potentially enhance vaccine efficacy (6).

In this context, dietary methionine supplementation has gained significant attention over the past decades (9–17). Methionine, an essential sulfur-containing amino acid, participates in multiple pathways with implications for health (18, 19). Beyond its essential role in protein synthesis, it acts as a precursor for S-adenosylmethionine (SAM), the principal methyl donor in a wide array of methylation reactions within the transmethylation pathway. Methionine also contributes to polyamine biosynthesis, which is critical for cell growth and differentiation, and serves as a substrate for cysteine synthesis via transsulfuration pathway, ultimately leading to the production of glutathione, a major antioxidant (18).

Furthermore, studies in European seabass (*Dicentrarchus labrax*) and rainbow trout (*Oncorhynchus mykiss*) have consistently reported increased peripheral leukocytes numbers, particularly neutrophils, in fish fed methionine-supplemented diets (12, 14), suggesting a role in immune cell proliferation and recruitment. Complementary *in vitro* research with primary head-kidney leukocytes isolated from European seabass further demonstrated the effects of methionine on enhancing cell viability, stimulating polyamine production and augmenting responsiveness to inflammatory stimuli (15), highlighting its capacity not only to increase immune cell numbers but also to prime them for a more effective response. Additional *in vitro* studies focusing on adaptive immunity have provided compelling evidence for methionine role in improving the survival of splenic leukocytes from rainbow trout and increasing the number of IgM-secreting cells (20). These findings directly implicate methionine in enhancing key aspects of cellular and humoral immunity. *In vivo* feeding trials have validated these effects, showing that adequate dietary methionine intake significantly increases circulating IgM-secreting cell numbers and enhances specific IgM antibody responses following antigen exposure (20). Taken together, these studies strongly suggest that methionine supplementation positively modulates both innate and adaptive immune mechanisms in fish. However, similar to observations in mammals (19), supplementation beyond physiological requirements can also exert detrimental effects on fish health. Indeed, several studies have shown that both immune and antioxidant defenses may plateau or even decline at high methionine intake (17, 20–26), emphasizing the need for careful selection of supplementation levels.

Although nutritional factors are known to have major effects on the immune response of fish, few studies have combined immunization with nutrient supplementation (6, 27), including amino acids (28, 29). Among these, Montero et al. (27) reported that vaccinated European seabass fed diets enriched with certain phytogenics showed higher survival rates following *Vibrio anguillarum* challenge compared to vaccinated fish fed a control diet. Although not involving a bacterial challenge, studies by Pohlenz et al. (28) and Peixoto et al. (29) demonstrated that dietary supplementation with individual amino acids, specifically arginine, glutamine or tryptophan, improved several immune parameters in vaccinated fish, such as higher antibody titers, suggesting enhanced immune responsiveness.

In this context, methionine emerges as a promising candidate to enhance vaccine efficacy, given its documented role in promoting both neutrophil and macrophage trafficking and proliferation, which may in turn enhance antigen uptake and presentation to lymphocytes. Therefore, it is hypothesized that methionine could act similarly to adjuvants classified as signal 1 facilitators by improving the immune-availability of vaccine antigens required for the activation of specific T and B lymphocytes (30). However, to the best of our knowledge, no studies have directly evaluated the effect of dietary methionine supplementation on vaccine efficacy in fish. Therefore, the present study aimed to investigate the immunomodulatory effects of dietary methionine supplementation in rainbow trout, focusing on its impact on the immune response and vaccine efficacy against the worldwide economically important pathogen *Yersinia ruckeri*, the causative agent of enteric redmouth disease (ERM) (31).

## 2 Materials and methods

### 2.1 Dietary treatments

Two diets differing in methionine content were formulated and manufactured by SPAROS Lda. (Olhão, Portugal) (**Table 1**). The control (CTRL) diet was designed to meet the indispensable amino acid requirements for rainbow trout (32) and, the second diet was identical to the CTRL diet but supplemented with DL-methionine (MET) at the expense of wheat gluten. Methionine concentrations in the CTRL and MET diets were 8 mg and 17.8 mg per gram of dry matter, respectively (**Table 2**). The methionine level in the MET diet was within the range previously reported to enhance immune function in rainbow trout (14).

**Table 1:** Ingredients and chemical composition of the experimental diets.

Ingredients	CTRL	MET
	%	
Fishmeal Super Prime^1^	20.00	20.00
Poultry meal^2^	7.50	7.50
Poultry blood meal^3^	2.00	2.00
Soy protein concentrate^4^	18.00	18.00
Wheat gluten^5^	12.00	11.00
Corn gluten meal^6^	8.00	8.00
Wheat meal^7^	9.90	9.90
Faba beans (low tannins)^8^	5.00	5.00
Vitamin and mineral premix^9^	1.00	1.00
Antioxidant^10^	0.20	0.20
Monocalcium phosphate^11^	1.00	1.00
Fish oil^12^	5.00	5.00
Rapeseed oil^13^	10.40	10.40
DL-Methionine^14^	0	1.00
Total	100	100
Proximate analysis (% dry weight)		
Phosphor (g/100g)	1.05	1.01
Ash (g/100g)	7.85	7.83
Energy (kJ/g)	22.32	22.43
Fat (g/100g)	19.12	19.19
Protein (g/100g)	50.75	50.97

^1^Fishmeal Super Prime: 66.3% CP, 11.5% CF, Pesquera Diamante, Peru.

^2^Poultry meal: 62.4% CP, 14.5% CF, SAVINOR UTS, Portugal.

^3^Poultry blood meal: 90% CP, 1% CF, ECB COMPANY SRL A S.U, Italy.

^4^Soycomil P: 63% CP, 0.8% CF, ADM, the Netherlands.

^5^VITAL: 83.7% CP, 1.6% CF, ROQUETTE Frères, France.

^6^Corn gluten meal: 61% CP, 6% CF, COPAM, Portugal.

^7^Wheat meal: 10.2% CP, 1.2% CF, Casa Lanchinha, Portugal.

^8^Faba beans: 28% CP, 1.2% CF, Ribeiro & Sousa Lda., Portugal.

^9^Vitamins (IU or mg/kg diet): dl-alpha tocopherol acetate, 100 mg; sodium menadione bisulphate, 25 mg; retinyl acetate, 20,000 IU; dl-cholecalciferol, 2000 IU; thiamin, 30 mg; riboflavin, 30 mg; pyridoxine, 20 mg; cyanocobalamin, 0.1 mg; nicotinic acid, 200 mg; folic acid, 15 mg; ascorbic acid, 500 mg; inositol, 500 mg; biotin, 3 mg; calcium panthotenate, 100 mg; choline chloride, 1000 mg, betaine, 500 mg. Minerals (g or mg/kg diet): copper sulphate, 9 mg; ferric sulphate, 6 mg; potassium iodide, 0.5 mg; manganese oxide, 9.6 mg; sodium selenite, 0.01 mg; zinc sulphate, 7.5 mg; sodium chloride, 400 mg; excipient wheat middlings), PREMIX Lda., Portugal.

^10^Paramega PX, Kemin Europe NV, Belgium.

^11^Monocalcium phosphate (22% phosphorus, 16% calcium), Fosfitalia, Italy.

^12^SAVINOR UTS, Portugal.

^13^Henry Lamotte Oils GmbH, Germany.

^14^DL-Methionine for Aquaculture (99% Methionine), Evonik Nutrition & Care GmbH, Germany.

**Table 2:** Amino acid composition (mg/g DW) of the experimental diets.

Amino acids	CTRL	MET
	mg AA/g DW diet	
Alanine	26.0	26.0
Arginine	30.0	29.2
Aspartic Acid + Asparagine	42.0	43.4
Glutamic Acid + Glutamine	92.0	95.7
Glycine	27.0	32.1
Histidine	11.0	10.6
Isoleucine	19.0	20.0
Leucine	41.0	36.5
Lysine	26.0	28.8
Phenylalanine	23.0	22.3
Proline	34.0	33.0
Serine	23.0	23.5
Threonine	18.0	18.4
Tyrosine	17.0	14.7
Valine	22.0	22.5
Methionine + Cystine	14.0	23.8
Cystine	6.0	6.0
Methionine	**8.0**	**17.8**

Methionine final concentration (mg methionine/g DW diet) in the two experimental diets is highlighted in bold.

Proximate composition of the experimental diets (**Table 1**) was analyzed following the procedures described in Carvalho et al. (26). Briefly, dry matter was determined by drying samples at 105°C for 24 h, and ash content was obtained after combustion at 550°C for 12 h. Crude protein (N × 6.25) was quantified using a flash combustion method coupled with gas chromatographic separation and thermal conductivity detection (LECO FP428). Crude fat was analyzed by petroleum ether extraction using the Soxhlet method, and gross energy was determined with an adiabatic bomb calorimeter (IKA).

### 2.2 Bacterial inoculum preparation

*Y. ruckeri* (strain QSP57.1, serovar I), isolated from rainbow trout in Portugal by Professor Alicia Toranzo (University of Santiago de Compostela, Santiago de Compostela, Spain), was cultured into tryptic soy agar (TSA, 1.5% NaCl; Pronadisa) plates overnight at 25°C. Individual colonies were then transferred to conical flasks containing 200 mL of tryptic soy broth (TSB, 1.5% NaCl; Difco), filling approximately two-fifth of the flask volume to ensure sufficient aeration, and cultured for 24 h under continuous agitation (130 rpm) at 25°C. After incubation, the bacterial concentration was measured at 600 nm and adjusted to 5 × 10^5^ colony-forming units (CFU)/mL. The concentration was confirmed by plating the resulting suspension on TSA plates and counting the colonies.

### 2.3 Experimental design

**Figure 1:**
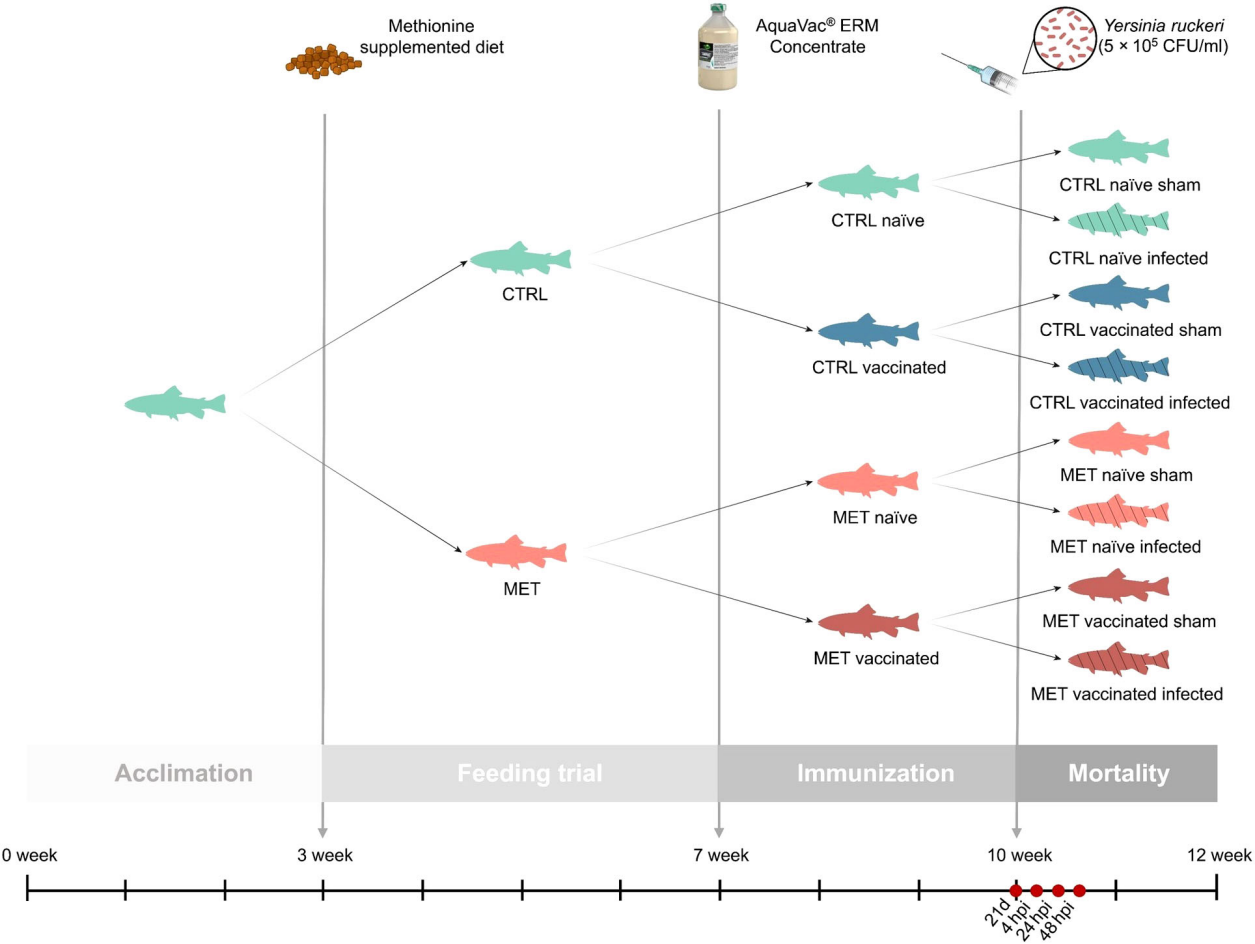
Experimental design.

Juvenile rainbow trout were transferred from a commercial fish farm (Serra de Aire, Portugal) to the CIIMAR facilities (Portugal), where they were housed for a period of eight weeks in a 2,000 L tank at 16°C and fed the CTRL diet twice a day at a rate of 4% body weight. After this period, a total of 1,120 fish (5.9 ± 0.9 g) were evenly distributed across 32 tanks (n = 35) of 60 L, within a semi-open recirculating freshwater system (water renewal rate: 60 L/h; temperature: 14 ± 0.5°C; pH: 6.99 ± 0.18; dissolved oxygen: 7.46 ± 0.54 mg/L; photoperiod: 12 h light/12 h dark). Following a three-week acclimation period, the feeding trial began with half of the tanks receiving the MET diet, while the other half continued on the CTRL diet. The feeding trial lasted four weeks, with fish being fed twice daily at a rate of 3% of their body weight (**Figure 1**).

At the end of the four-week feeding period, the water temperature was gradually increased by 1°C per day for two days, reaching 16 ± 0.5°C. Subsequently, half of the fish within each dietary group were dip-vaccinated against *Y. ruckeri* with AquaVac^®^ ERM Concentrate (MSD Animal Health), while the other half were sham-vaccinated to serve as control (**Figure 1**). Following the manufacturer’s instructions, fish were immersed in a solution of 1 L of vaccine diluted with 9 L of well-aerated water for 30 s, after which they were returned to their original tanks. To achieve the 336 degree-days stipulated in the product information, the water temperature was kept at 16 ± 0.5°C throughout the three-week immunization period. During this period, fish continued to be fed their respective experimental diets.

Twenty-one days post-vaccination (21d), one fish per tank (n = 8) was sampled to assess the potential synergistic effects of the vaccine and dietary treatment on immune status. The remaining fish were intraperitoneally challenged with 100 µL of the previously prepared *Y. ruckeri* inoculum (5 x 10^5^ CFU/mL) or injected with Hanks’ Balanced Salt Solution (HBSS) as a sham group. The inoculum concentration was selected based on preliminary challenge trials. Water temperature was increased to 17.5 ± 0.5°C, a temperature close to the optimal for *Y. ruckeri* infectivity (33), and maintained at this level until the end of the trial. At this stage, fish were divided into two systems: one for mortality monitoring and the other for a time-course trial. For mortality monitoring, 10 fish per tank were retained in the initial system (n = 40), while for the time-course trial, 30 infected fish per treatment were transferred to a similar separate system (10 fish per tank in triplicate tanks). Daily mortality was monitored over 13 days and cumulative survival was calculated accordingly. The time-course trial consisted of three sampling points, particularly at 4, 24 and 48 h post-infection (hpi) (n = 9 at each time) (**Figure 1**).

All procedures were conducted by trained scientists in accordance with FELASA category C recommendations, and complied with national regulations, as well as the European Directive 2010/63/EU on the protection of animals used for scientific purposes.

### 2.4 Sampling procedures

To evaluate the effects of methionine supplementation on the immune status and immune response to *Y. ruckeri* in both vaccinated and naïve rainbow trout, four sampling times were defined: one after the four-week feeding trial and 21 days post-vaccination (21d), and three post-infection, particularly at 4, 24 and 48 hpi (**Figure 1**). At the first sampling point (21d) one fish per tank (n = 8) was euthanized as described below and for each subsequent post-infection time point, three infected fish per tank were sampled (n = 9).

Fish were euthanized using a solution of water with 2-phenoxyethanol (0.5 mL/L; Sigma-Aldrich). Immediately following blood collection from the caudal vessel, a portion of the sample was used for *in situ* counting of circulating red blood cells (RBC) and white blood cells (WBC) per microliter of blood using an improved Neubauer chamber, as well as for hematocrit measurement. Blood smears were also prepared, air-dried, and used for leukocyte counting and categorization. The remaining blood was centrifuged (10 min, 10,000 × *g*, room temperature) and plasma collected, flash-frozen in liquid nitrogen and stored at −80°C until proteomic analysis. Gills, posterior gut and spleen were collected and stored in RNAlater (1:10 w/v; Sigma-Aldrich) at 4°C for the first 24 h and then transferred to −80°C until *Y. ruckeri* quantification by RT-PCR was performed. The liver was collected and immediately frozen at −80°C for metabolite analysis, oxidative stress biomarkers evaluation and gene expression analysis. The head-kidney was also collected and stored in RNA later in the same conditions as described above for gene expression analysis.

### 2.5 Analytical procedures

#### 2.5.1 Hematological profile

Along with total circulating WBC and RBC counts and hematocrit measurement, hemoglobin levels were determined using a SPINREACT kit (ref. 1001230). Mean corpuscular hemoglobin (MCH), mean corpuscular volume (MCV) and mean corpuscular hemoglobin concentration (MCHC) were calculated as described by Machado et al. (11).

#### 2.5.2 Differential peripheral leukocytes count

Blood smears were fixed using a solution of 10% formaldehyde (37%) in absolute ethanol. Neutrophils were stained using the technique described by Afonso et al. (34), followed by Wright’s stain (Hemacolor, Merck). Slides were examined under oil immersion (1,000×). For each slide, two hundred leukocytes were classified into thrombocytes, lymphocytes, monocytes and neutrophils. The percentage of each leukocyte type was calculated and multiplied by the total number of WBC to determine the number of cells per mL.

#### 2.5.3 Quantification of bacterial load

*Y. ruckeri* was quantified in three tissues: gills, posterior gut and spleen, following the methodology described by Bastardo et al. (35). Briefly, from the 8 fish per treatment sampled before infection, DNA was extracted from a randomly selected subset of 3 fish per treatment to serve as negative controls, and from 9 fish per treatment at each time post-infection, using the Maxwell^®^ RSC PureFood GMO and Authentication Kit (Promega Corporation) following the manufacturer’s instructions. DNA integrity was assessed by gel electrophoresis and its purity and concentration were measured using a DS-11 FX spectrophotometer (DeNovix). The DNA concentration was then adjusted to 22.5 ng/µL, ensuring approximately 100 ng of template per reaction for the subsequent real-time PCR assay.

The assay targeted the *recA* gene (recombination protein A), used species-specific primers (YR-F, 5’-TCTGGACATCGCTCTGG-3’, and YR-R, 5’-AGTTTTTTTGCGTAGATAGGA-3’) and a probe (5’-[FAM] TATCGCCTCTGCACAGC [MGBEQ]-3’) (Eurofins Genomics) (35). Each reaction consisted of 4.45 μL of diluted DNA mixed with 5 μL of iTAQ Universal Probes Supermix (BioRad), 0.2 μL (10 μM) of each primer and 0.15 μL of probe, in a final volume of 10 μL. Thermal cycling conditions included an initial denaturation at 95°C for 10 min, followed by 40 cycles of three steps: 95°C for 30 s, 62°C for 35 s and 72°C for 1 min. All reactions were carried out with technical duplicates using a CFX384 Touch Real-Time PCR Detection System (Biorad).

To determine bacterial load, a standard curve was generated using tenfold serial dilutions of DNA obtained from a *Y. ruckeri* culture with an initial concentration of 2.1 × 10^9^ CFU/mL, as confirmed by colony count. The detection limit was defined as the lowest concentration within the linear range that produced an amplification signal. Sample cycle thresholds (Ct) values obtained by RT-PCR were interpolated on the standard curve to calculate *Y. ruckeri* concentration in the tissues (CFU/mL) (35).

#### 2.5.4 Liver samples preparation

To evaluate metabolite levels, antioxidant capacity and gene expression, liver samples were collected from 8 fish per treatment before infection and from 9 fish per treatment at each time post-infection. Each liver sample was then divided into three portions on dry ice. Following the procedure described by Peixoto et al. (36), one portion was weighed and homogenized by ultrasonic disruption in 7.5 volumes of ice-cold 0.6 N perchloric acid. The homogenate was neutralized with 1 M potassium bicarbonate and centrifuged (1,359 × *g*, 30 min, 4°C). The resulting supernatant was used for metabolite quantification. A second liver portion, for antioxidant capacity analysis, was weighed and homogenized in K-phosphate buffer (0.1 M, pH = 7.4) at a 1:10 (w/v) ratio. After sonication, an aliquot was taken for lipid peroxidation (LPO) analysis, mixed with 4% BHT (2,6-Di-tert-butyl-4-methylphenol) in methanol and immediately frozen at −80°C. The remaining homogenate was centrifuged (10,000 × *g*, 20 min, 4°C) and the supernatants were divided into aliquots and stored to conduct antioxidant capacity assays. The third portion was homogenized in 200 µL of 1-thioglycerol/homogenization solution (Maxwell^®^ RSC simplyRNA Tissue Kit, Promega Corporation) using Precellys Evolution tissue-lyser homogenizer.

##### 2.5.4.1 Hepatic metabolites

The hepatic metabolite quantification was performed as described in Peixoto et al. (36). Briefly, lactate, triglycerides (TAG), cholesterol and glucose levels were determined spectrophotometrically using commercial kits (Spinreact 1001330, 1001311, 1001090 and 1001200, respectively). Tissue glycogen concentration was assessed as described by Keppler and Decker (37). For that, tissue homogenates were incubated at 37°C for 2 h, with and without amyloglucosidase (Sigma-Aldrich), which hydrolyzes glycogen into glucose. Glycogen content was then calculated as glucose equivalents after subtracting the free glucose concentration. All analyses were conducted in duplicate and absorbances were measured on a Synergy HT microplate reader (Biotek) at 505 nm for lactate, TAG and cholesterol, and 340 nm for glucose and glycogen.

##### 2.5.4.2 Antioxidant responses

Protein concentration in liver homogenates was determined using the Pierce BCA Protein Assay Kit (Thermo Scientific) as described by Peixoto et al. (38). Protein concentration and all subsequent antioxidant capacity protocols were performed in triplicate and absorbances were measured using a Synergy HT microplate reader (Biotek).

LPO levels were assessed by quantifying malondialdehyde (MDA), a byproduct of LPO that reacts with thiobarbituric acid (TBA) to form a pink chromogen measurable at 535 nm (39). Liver homogenates (204 µL) were mixed with 100 µL of cold trichloroacetic acid (TCA, 100%) and 1 mL of a 0.73% TBA solution prepared in 60 mM Tris–HCl (pH = 7.4) with 0.1 mM diethylenetriaminepentaacetic acid (DTPA). The mixture was incubated at 100°C for 1 hour, centrifuged (15,000 × *g*, 5 min, room temperature), and 200 µL of the supernatant transferred to a 96-well microplate. Absorbance was measured at 535 nm, with ultrapure water as negative control. Results were expressed as nanomoles of thiobarbituric acid reactive substances (TBARS) or MDA equivalents per gram of wet tissue, calculated from a standard calibration curve.

Catalase (CAT; EC 1.11.1.6) activity was determined by measuring the decrease in hydrogen peroxidase concentration over time (40). Liver homogenates were diluted to 0.7 mg/mL in K-phosphate buffer (0.1 M, pH = 7.4). Briefly, 10 µL of the diluted sample, 140 µL of K-phosphate buffer (0.05 M, pH = 7.0) and 150 µL of hydrogen peroxide solution (30% H_2_O_2_ in K-phosphate buffer, 0.05 M, pH = 7.0) were added to a 96-well UV microplate. Absorbance was recorded at 240 nm for 1 min every 15 s. The negative control consisted of K-phosphate buffer (0.1 M, pH = 7.4) instead of the sample. CAT activity was expressed as enzymatic units (U/mg protein), where one unit is defined as the amount of enzyme required to transform 1 µmol of substrate per minute.

Superoxide dismutase (SOD; EC 1.15.1.1) activity was measured using a spectrophotometric assay to evaluate the capacity of SOD to compete with ferricytochrome C for O_2_•^−^ radicals generated by the xanthine/xanthine oxidase system (41). Liver homogenates were diluted with K-phosphate buffer (0.1 M, pH = 7.4) to a concentration of 0.3 mg/mL, and then mixed with 200 µL of a solution containing 50 mM potassium phosphate buffer (pH = 7.8), 0.1 mM NaEDTA, 0.7 mM xanthine and 0.03 mM cytochrome C, followed by the addition of 50 µL of xanthine oxidase (0.03 U/mL). Absorbance was read at 550 nm every 20 s for 3 min. A standard curve was created through serial dilutions of a stock solution of SOD. Results were expressed as U/mg protein, with one unit defined as the enzyme amount required to achieve 50% inhibition of ferricytochrome C reduction.

Total glutathione (tGSH), reduced glutathione (rGSH) and oxidized glutathione (GSSG) concentrations were measured using the GSH/GSSG Microplate Assay Kit (Oxford Biomedical Research) as described by Hamre et al. (42). tGSH was measured by reacting all available rGSH and GSSG with 5,5’-dithiobis-2-nitrobenzoic acid (DTNB), generating a yellow-colored product measurable at 412 nm. For GSSG quantification, a thiol scavenger (N-ethylmaleimide pyridine derivative) was added to bind and remove rGSH, leaving only GSSG. The GSSG was then reduced using glutathione reductase, and this newly formed rGSH reacted with DTNB to quantify the original GSSG concentration. The rGSH/GSSG ratio was calculated using the formula: (tGSH – 2 × GSSG)/GSSG.

#### 2.5.5 Gene expression

Total RNA was extracted from liver and head-kidney using the Maxwell^®^ RSC simplyRNA Tissue Kit (Promega Corporation) according to the manufacturer’s specifications. RNA concentration was quantified with a DS-11 FX spectrophotometer (DeNovix). First-strand complementary DNA (cDNA) was synthesized using the NZY First-Strand cDNA Synthesis Kit (NZYTech), with reactions performed on a Veriti DX 96-well Thermal Cycler (Applied Biosystems). Quantitative real-time PCR (qPCR) was conducted using a CFX384 Touch Real-Time PCR Detection System (Biorad). Reactions were prepared in a final volume of 10 µL, containing 4.4 µL of diluted cDNA, 5 µL of NZYSupreme qPCR Green Master Mix (NZYtech) and 0.3 µL (10 µM) of each primer. The reactions were set up in 384-well plates. Primers were designed with NCBI Primer-BLAST Tool, adhering to standard qPCR design criteria. Primer efficiency was determined using serial 2-fold dilutions of randomly pooled cDNA by calculating the slope of the regression line of the Ct values. Melting curve analysis was performed to ensure the absence of primer dimers. Standard cycling conditions included an initial denaturation at 95°C for 10 min, followed by 40 cycles of denaturation at 95°C for 15 s, annealing at the primer-specific temperature for 1 min and extension at 72°C for 1 min. All reactions were run in duplicate and gene expression levels were normalized using the geometric mean of elongation factor 1-alpha (*ef1a*) and beta-actin (*bact*) and calculated according to Pfaffl method (43). A set of primers was designed to target genes involved in different biological processes, including the methionine-cycle (betaine-homocysteine methyltransferase [*bhmt*], S-adenosylhomocysteine hydrolase-like protein 1 [*sahh1*], S-adenosylmethionine decarboxylase proenzyme-like [*amd1*], spermidine/spermine N1-acetyl transferase 1 [*sat1*] and spermine synthase [*sms*]), energy metabolism (glutamate dehydrogenase [*gdh*] and hydroxyacyl-coenzyme A dehydrogenase [*hoad*]), innate immunity and inflammation (interleukins 1 beta [*il1b*], 8 [*il8*] and 10 [*il10*], liver-expressed antimicrobial peptide 2 [*leap2*], serum amyloid A [*saa*], toll-like receptors 1 [*tlr1*] and 5 [*tlr5*], transforming growth factor beta [*tgfb*] and tumor necrosis factor alpha [*tnfa*]), T cells markers (clusters of differentiation 4 [*cd4*], 8 alpha chain [*cd8a*] and 8 beta chain [*cd8b*], and major histocompatibility complex class I [*mhci*]) and antibody markers (immunoglobulins M heavy chain [*igm*], immunoglobulin T [*igt*] and membrane immunoglobulin D [*igd*]). Detailed information on primer sequences, amplification efficiencies, annealing temperatures, amplicon lengths, and accession numbers is provided in **Table 3**.

**Table 3:** Oligonucleotides used for real-time PCR.

Gene	Acronym	Accession number	Efficiency	AT (°C)	Amplicon length	Primer sequence (5’ – 3’)
Elongation factor 1-alpha	*ef1a*	NM_001124339.1	98.61	60	150	F: GATCCAGAAGGAGGTCACCA R: TTACGTTCGACCTTCCATCC
Beta-actin	*bact*	NM_001124235.1	104.56	60	105	F: GATGGGCCAGAAAGACAGCTA R: TCGTCCCAGTTGGTGACGAT
Betaine-homocysteine methyltransferase	*bhmt*	XM_021602136.2	103.09	60	292	F: CCGAAGCGGTTGGTTGAGAA R: TTATCGTCGCTGGCGTAGAA
Cluster of differentiation 4	*cd4*	AY973028.1	113.11	60	182	F: GTTCTTGATCATTGGCGAGGG R: CCCCACTTTGATCTGGGGAG
Cluster of differentiation 8 alpha chain	*cd8a*	AF178054.1	97.29	60	202	F: CGACGACTACACCAATGACC R: TGTGGGCATCTTTTTGTTCTT
Cluster of differentiation 8 beta chain	*cd8b*	NM_001124008.1	118.39	60	131	F: GTTCAAGGCCAGTAAAAGGGACAT R: GCCTCCACAACTCGTTCTCTTTCT
Glutamate dehydrogenase	*gdh*	AJ41957.1	125.13	60	140	F: ATCAAGCCCTGCAACCACGTCCT R: CTTCACTGTAACGGATCCCCCCTTT
Hydroxyacyl-coenzyme A dehydrogenase	*hoad*	XM_021573383.2	131.35	60	126	F: GGACAAAGTGGCACCAGCAC R: GGGACGGGGTTGAAGAAGTG
Immunoglobulin M heavy chain	*igm*	S63348.1	100.69	60	155	F: ATTTGAATGCGCCGTGGAAC R: CATTGGCAAAGCAGGCGAAG
Immunoglobulin T	*igt*	XM_036941448.1	101.85	60	80	F: AACATCACCTGGCACATCAA R: TTCAGGTTGCCCTTTGATTC
Interleukin 1 beta	*il1b*	AJ278242.2	109.06	58	62	F: CGTCACTGACTCTGAGAACAAGT R: TGGCGTGCAGCTCCATAG
Interleukin 8	*il8*	XM_021625343.1	116.89	60	120	F: AGAGACACTGAGATCATTGCCAC R: CCCTCTTCATTTGTTGTTGGC
Interleukin 10	*il10*	NM_001245099.1	101.08	58	277	F: CTGCTGGACGAAGGGATTCTAC R: GGCCTTTATCCTGCATCTTCTC
Liver-expressed antimicrobial peptide 2	*leap2*	NM_001124464.1	112.12	60	222	F: GGTTCCTGGTGTTTCTGGTGCT R: AGTGGCCACCCCTGCAAAT
Major histocompatibility complex class I	*mhci*	EU036638.1	128.89	60	91	F: GCAACCCAATTTCATGCAGG R: ACACTCAATGCAGGTCTGGG
Membrane immunoglobulin D	*migd*	XM_036941453.1	98.28	60	138	F: CAGGAGGAAAGTTCGGCATCA R: CCTCAAGGAGCTCTGGTTTGGA
S-adenosylhomocysteine hydrolase-like protein 1	*sahh1*	XM_021569325.2	100.62	60	141	F: TGTCTGCATTGATGTCGCTCT R: GGGTATACACATGGTCCAGC
S-adenosylmethionine decarboxylase proenzyme-like	*amd1*	XM_021611778.1	110.17	60	162	F: ACCTCCGTACCATCCCAAG R: TGGTTCCACACGTCTTCAAA
Serum amyloid A	*saa*	NM_001124436.1	110.14	58	157	F: GACGCCAACTGGAAAAACTC R: CTGCTGAGTCCTCGTGTCCT
Spermidine/spermine N1-acetyl transferase 1	*sat1*	NM_001160572.1	115.2	60	283	F: TCATGGAAGAGTACAGGGGGT R: TAGCCTTAGTCTCTCACTCCCATC
Spermine synthase	*sms*	XM_021618707.1	117.12	57	105	F: TGTTGGTGGAGGACTGTGTT R: GCGCTGTGGAGATAGGAACT
Toll-like receptor 1	*tlr1*	NM_001166101.1	130.51	60	90	F: CAGACGCCCTGTTGATGTTC R: CCTTCACAAGTTCCACCACG
Toll-like receptor 5	*tlr5*	NM_001124208.1	100.62	60	135	F: TTGACTTATCTTCCAACGGATTCA R: CTTTGAAATTGCTGAAACCAAATG
Transforming growth factor beta	*tgfb*	XM_021591332.1	106.41	58	229	F: AGCTCTCGGAAGAAACGACA R: CGGGGTTGTGGTGCT
Tumor necrosis factor alpha	*tnfa*	NM_001124374.1	117.88	60	66	F: GGGGACAAACTGTGGACTGA R: GAAGTTCTTGCCCTGCTCTG

AT, Annealing Temperature.

#### 2.5.6 Proteomic analysis

##### 2.5.6.1 Sample preparation

Plasma samples from five fish per treatment, collected before infection (21d) and at 24 and 48 hpi, were randomly selected and sent for proteomic analysis (Aalborg University, Denmark), resulting in a total of 60 samples. Sample preparation for mass spectrometry (MS) followed the filter-aided sample preparation (FASP) method (44). Briefly, 100 µg of total protein from each sample was dissolved in 5% sodium deoxycholate in 50 mM triethylammonium bicarbonate (pH = 8.6). Proteins were reduced and alkylated with 10 mM Tris(2-carboxyethyl)phosphine hydrochloride and 25 mM chloroacetamide for 30 min at room temperature. Samples were then digested overnight at 37°C with sequencing grade trypsin (Promega) at 1:100 (w/w) enzyme-to-protein ratio. Peptides were extracted via solvent phase transfer, as described in Masuda et al. (45), and stored at –80°C until analyzed.

##### 2.5.6.2 Analytical platform and data acquisition

Quantitative label-free liquid chromatography-mass spectrometry (LC-MS) analyses were performed on an EvosepONE (Evosep Biosystems) coupled to a CaptiveSpray nanoelectrospray ionisation source (ZDV 20 nm emitter, Bruker) and a timsTOF Pro2 mass spectrometer (Bruker). Peptides were loaded onto Evotip Pure cartridges (Evosep Biosystems) using an automated OT-2 Evotip Loading Protocol (v1.3). For each sample, 200 ng of peptides were analyzed using the 60SPD LC-MS method, with quality control (QC) standards (200 ng Hela tryptic digest Thermo Scientific; Biognosys iRT) run between samples. The mass spectrometer was operated in positive ionization mode with data-independent acquisition and parallel accumulation-serial fragmentation (diaPASEF-long gradient) enabled. Sample QC analysis was automatically performed using the ProteoScape GPU based search engine.

##### 2.5.6.3 Data processing

Protein identification and quantification were performed in Spectronaut v19.5 (Biognosys) using directDIA+ with default settings (global normalization by median; LFQ QUANT2.0 MS1). Raw files were matched against the UniProt reference proteome for rainbow trout (proteome ID: UP000193380; protein count: 46,436). Decoy entries and proteins identified by fewer than two peptide sequences were excluded. All related and quantitative information (PG.Quantity) was exported in TSV format. The mass spectrometry proteomics data have been deposited to the ProteomeXchange Consortium via the PRIDE (46) partner repository with the dataset identifier PXD068409.

##### 2.5.6.4 Differential abundance analysis

Data were imported into Perseus (v2.1.3.0), missing values were imputed from a normal distribution to simulate signals of low-abundant proteins and principal component analysis (PCA) was performed to visualize the distribution of samples. With the exception of the CTRL vaccinated group at 24 hpi, one sample was removed from each of the other treatment groups. After outlier exclusion, data were log_2_-transformed and two-sample *t*-tests [Student’s *t*-test with Benjamini-Hochberg correction (FDR = 0.05)] were conducted to compare all experimental groups with the CTRL naïve group before infection, which was used as a common reference. Proteins with an adjusted *p*-value (*p*_adj_) < 0.05 were considered differentially abundant proteins (DAPs).

#### 2.5.7 Statistical analysis

Except for proteomic data, statistical analyses were conducted using IBM SPSS v29.0 Statistics for Windows. Data were tested for normality and homogeneity of variances using the Shapiro-Wilk and Levene’s tests, respectively. Outliers were removed and data transformed whenever necessary. A two-way ANOVA was performed to investigate the effects of diet and vaccination on the immune status of fish after four weeks on experimental diets and three weeks post-vaccination (21d). To explore the effects of diet, vaccination status and time post-challenge on the early immune response to *Y. ruckeri*, a three-way ANOVA was conducted. When significant interactions between factors were observed, additional ANOVA analyses were performed to assess the individual effects of specific factors. When time effects were significant, *post-hoc* comparisons were made using the Tukey’s test.

Chi-square tests were applied to investigate the effects of dietary treatment and vaccination status on cumulative survival, as well as to assess differences in the proportion *of Y. ruckeri*-positive fish across dietary groups, vaccination status, time points, tissues and the diet × vaccination interaction. When significant differences were detected, and to investigate differences between experimental groups within specific tissues and time points, pairwise comparisons were performed using Fisher’s exact test. Bacterial load data were analyzed using the Mann-Whitney *U* test or Kruskal-Wallis test, followed by Bonferroni-adjusted pairwise comparisons when appropriate.

For all the tests, 95% confidence interval (CI) was used, giving a probability level of 0.05. Data are presented as means ± standard deviation (mean ± SD). All plots were generated using the R v4.2.1/ggplot2 package v3.5.1.

## 3 Results

### 3.1 Immune status

After the 7-week feeding period (21d post-vaccination), only a few differences were detected across experimental groups. Vaccinated fish showed higher MCV levels and lower *cd8b* expression levels in the head-kidney compared to naïve fish (**Supplementary File S1**; **Supplementary Tables S1**, **S6**, respectively). In addition, MET-fed fish exhibited significantly lower hepatic mRNA expression of *amd1* than CTRL-fed fish, regardless of vaccination status (**Supplementary File S1**; **Supplementary Table S5**). No other differences were detected in the hematological profile or differential leukocyte counts across experimental groups (**Supplementary File S1**; **Supplementary Tables S1**, **S2**). Similarly, hepatic metabolites levels (**Supplementary File S1**; **Supplementary Table S3**) and antioxidant capacity (**Supplementary File S1**; **Supplementary Table S4**) did not differ significantly among groups.

### 3.2 Cumulative survival

**Figure 2:**
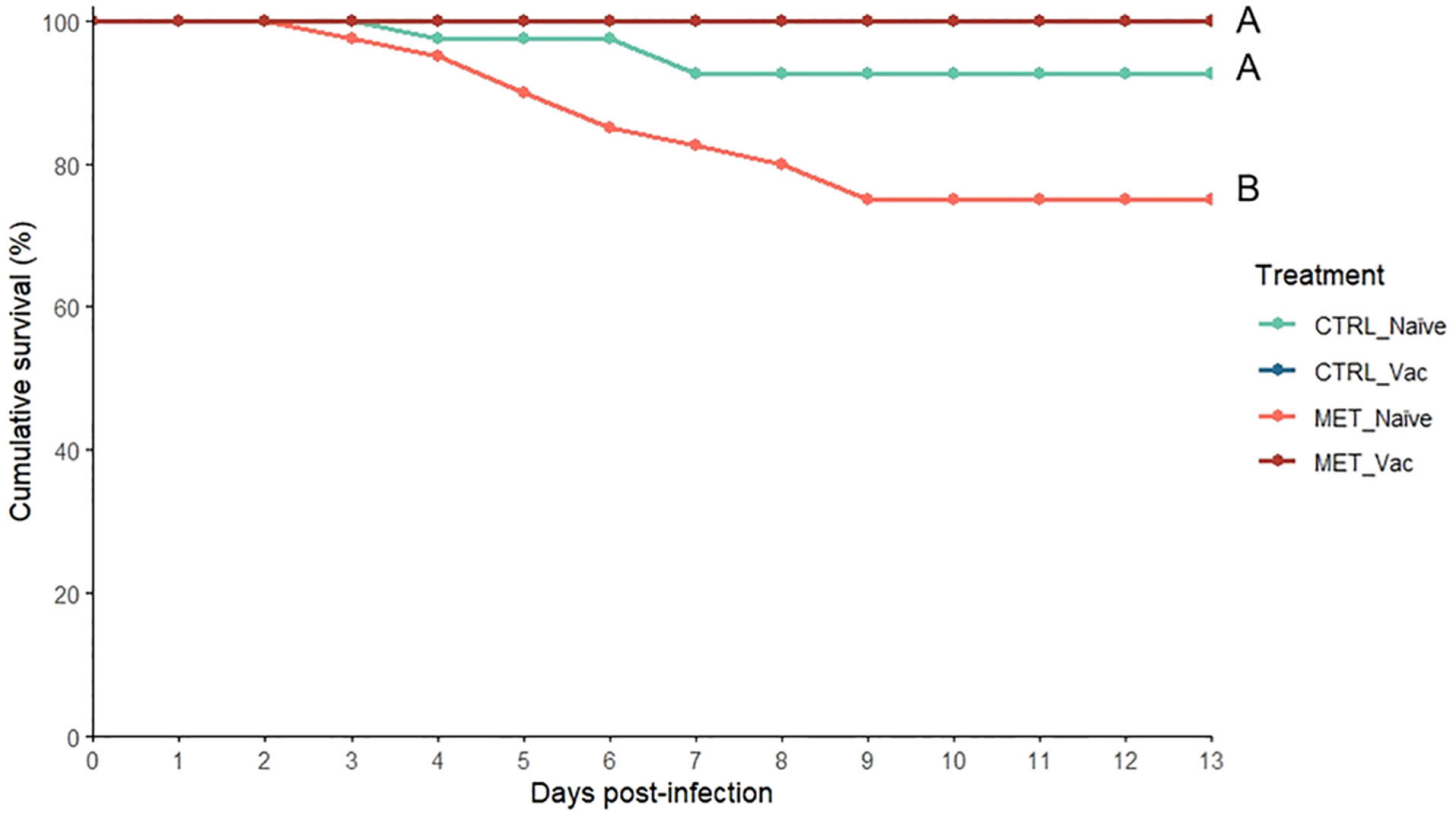
Cumulative survival of rainbow trout intraperitoneally infected with *Y. ruckeri* (5 × 10^5^ CFU/mL) following 7 weeks (4 weeks before vaccination and 3 weeks post-vaccination) of feeding with CTRL or MET diet (n = 40). Sham-infected fish are not shown for the clarity of the graph. Different letters denote differences across experimental groups (Fisher’s exact test; *p* ≤ 0.05).

All vaccinated fish survived the bacterial challenge. In contrast, cumulative survival in naïve fish began to decline at 3 days post-infection. By the end of the 13-day mortality trial, MET naïve fish had a markedly lower cumulative survival rate (75%) compared to the CTRL naïve group (92.5%) and both vaccinated groups (100%) (**Figure 2**). No mortality was observed in the sham-infected control group during this period (data not shown).

### 3.3 Bacterial load

**Figure 3:**
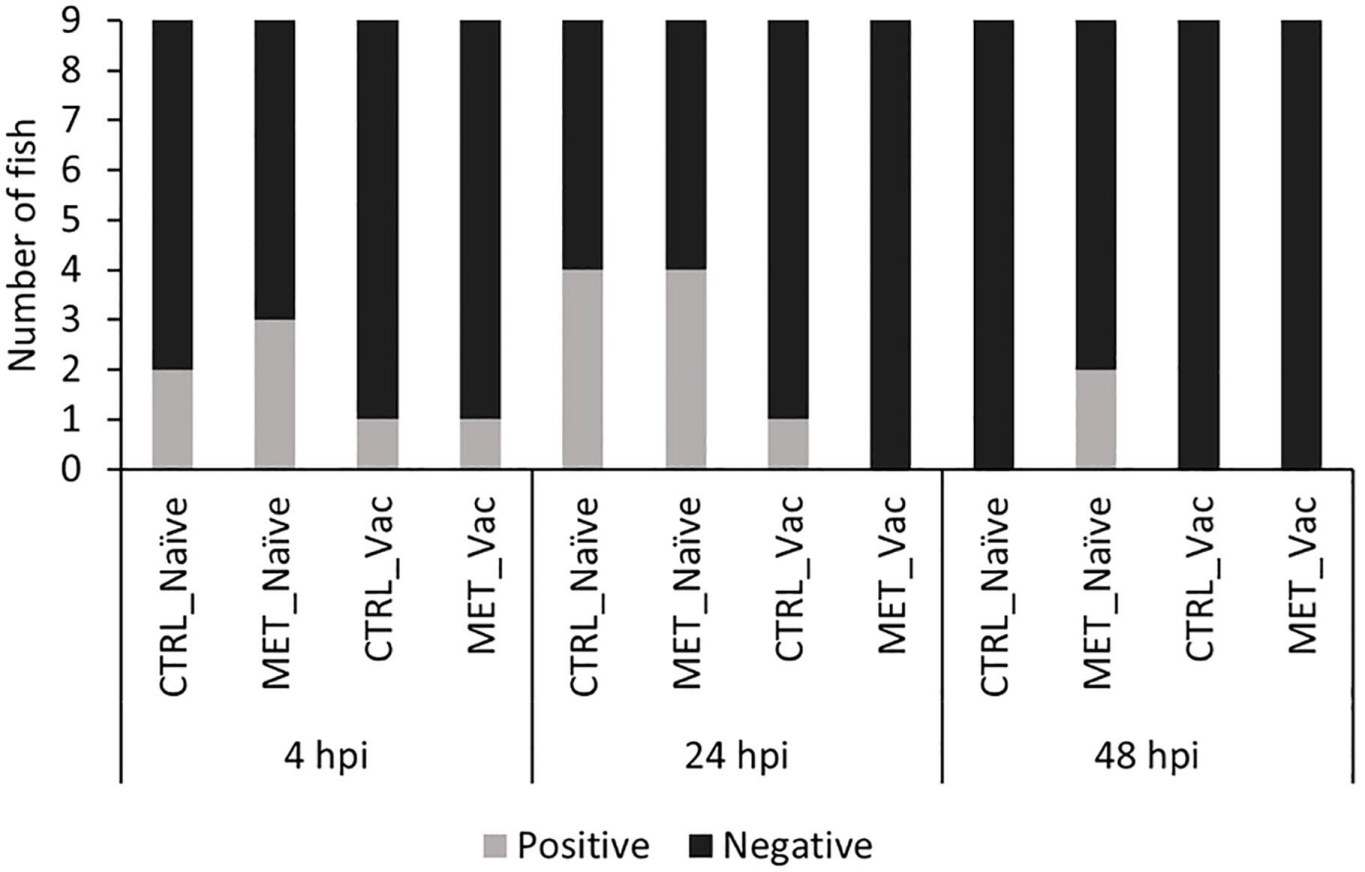
Number of rainbow trout with detectable *Y. ruckeri* in at least one of the three tissues analyzed (gills, posterior gut and spleen) at 4, 24 and 48 h post-infection infection. Fish were considered positive when bacterial load in any tissue was ≥ 230 CFU/mL.

**Figure 4:**
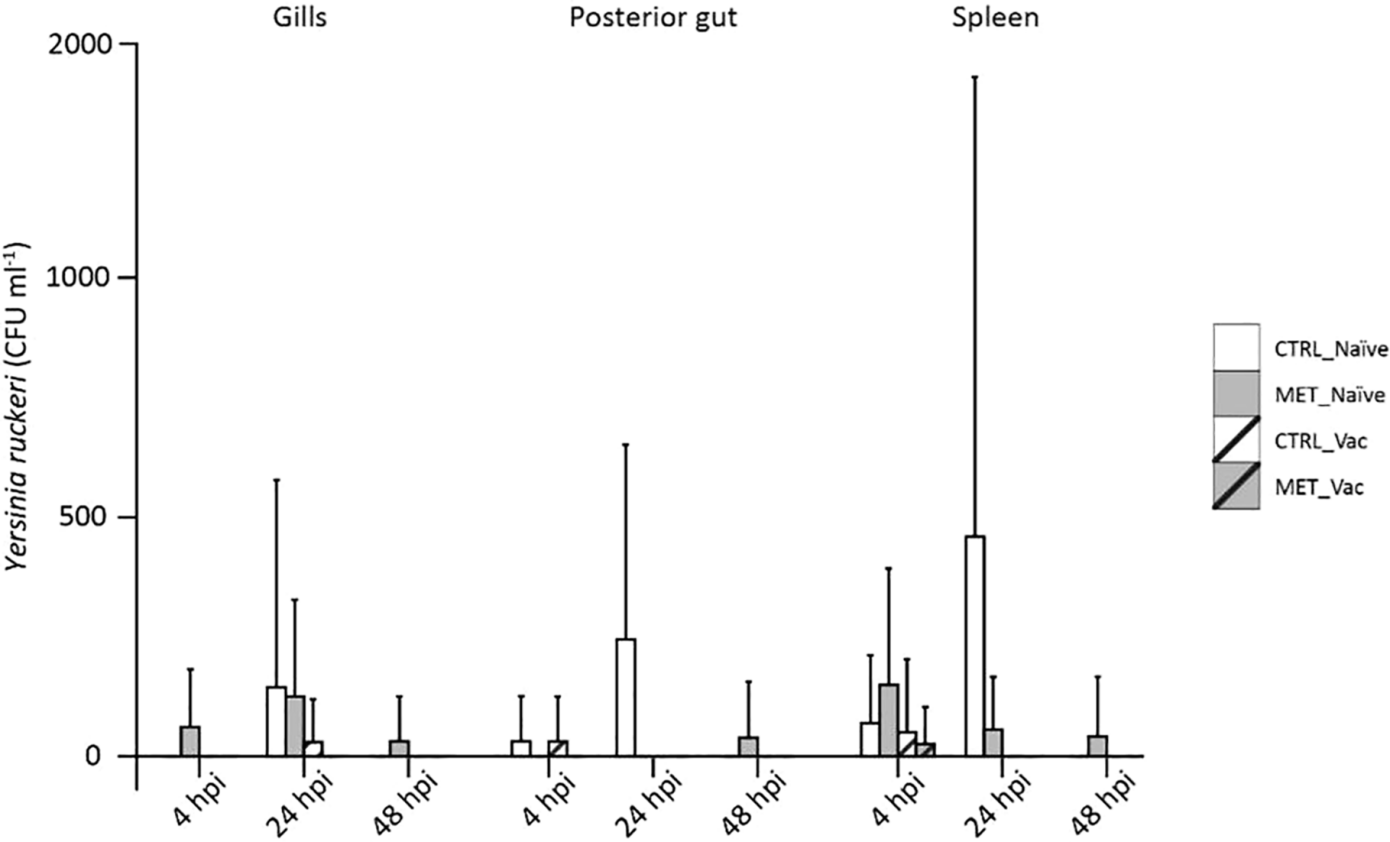
Concentration of *Y. ruckeri* in the gills, posterior gut and spleen of rainbow trout at 4, 24 and 48 h post-infection following 7 weeks (4 weeks before vaccination and 3 weeks post-vaccination) of feeding with CTRL or MET diets. Bacterial load is expressed as the mean concentration (CFU/mL) and standard deviation for each tissue and treatment group (n = 9).

Bacterial load was quantified in three tissues (gills, posterior gut and spleen) before infection (21d) and at three times post-infection (4, 24 and 48 hpi) across all four experimental groups (CTRL naïve, MET naïve, CTRL vaccinated, MET vaccinated). Both the number of fish with detectable *Y. ruckeri* (**Figure 3**) and the bacterial concentration in each tissue were assessed (**Figure 4**).

Prior to infection, *Y. ruckeri* was not detected in any experimental group (data not shown). Following infection, the number of fish carrying detectable bacteria in at least one analyzed tissue was higher in the MET-naïve group than in both vaccinated groups, although it did not significantly differ from the CTRL-naïve group (**Supplementary File S1**; **Supplementary Table S7**). While no statistically significant differences in bacterial prevalence were observed among groups when analyzed at individual time points (**Supplementary File S1**; **Supplementary Table S8**), at 48 hpi, the MET-naïve group was the only one in which *Y. ruckeri*-positive fish were still detected (**Figure 3**). The number of gills, posterior gut and spleen samples testing positive for *Y. ruckeri* is provided in **Supplementary File S1**; **Supplementary Figure S1**.

Bacterial load peaked at 24 hpi and decreased thereafter (**Supplementary File S1**; **Supplementary Table S9**). Consistent with the prevalence data, CTRL-naïve fish exhibited bacterial loads comparable to both vaccinated and MET-naïve groups, while MET-naïve fish consistently showed significantly higher bacterial concentrations than vaccinated fish, irrespective of tissue or time point (**Supplementary File S1**; **Supplementary Table S9**). At 24 hpi, CTRL-naïve fish displayed the highest *Y. ruckeri* concentration in the posterior gut when compared to all other groups (**Figure 4**; **Supplementary File S1**; **Supplementary Table S10**).

Overall, both bacterial prevalence and load were consistently higher in MET-naïve fish than in vaccinated groups, whereas CTRL-naïve fish showed values comparable to vaccinated fish.

### 3.4 Immune response to infection

#### 3.4.1 Hematological parameters and cell counts

While no differences were observed in the number of RBC following infection, the hematocrit was higher in vaccinated fish at 48 hpi, significantly surpassing the observed in naïve fish (**Supplementary File S1**; **Supplementary Table S11**). Conversely, at 24 hpi, naïve fish exhibited an increase in MCV compared to pre-infection levels. Hemoglobin and MCHC levels were also influenced by time, vaccination and diet. At 4 hpi, the CTRL vaccinated group had higher hemoglobin levels than both the CTRL naïve and MET vaccinated groups. At 24 hpi, the MET vaccinated group had higher hemoglobin levels than those in the CTRL vaccinated group. At 4 hpi, among the naïve fish, MCHC levels were higher in the MET-fed fish compared to CTRL-fed fish. In contrast, among vaccinated fish, CTRL-fed fish showed higher MCHC levels than MET-fed fish. Moreover, the MET vaccinated group had lower MCHC levels than MET naïve group. At 24 hpi, the CTRL vaccinated fish exhibited the lowest MCHC levels, which were significantly inferior than those observed in both CTRL naïve and MET vaccinated fish (**Supplementary File S1**; **Supplementary Table S11**).

A significant decrease in total WBC counts and circulating lymphocytes was observed across all groups post-infection. Furthermore, both parameters were affected by vaccination, with vaccinated fish displaying higher WBC counts and lymphocyte numbers than naïve fish. On the opposite, the number of circulating thrombocytes increased at 48 hpi compared to pre-infection values, the number of monocytes peaked at 24 hpi, before returning to lower levels at 48 hpi, and the neutrophil numbers were higher at 24 hpi compared to 4 hpi, though not significantly different from pre-infection values (**Supplementary File S1**; **Supplementary Table S12**).

In summary, although interactions between diet and vaccination were observed for hemoglobin and MCHC levels, dietary treatment did not markedly influence other hematological parameters or differential leukocyte counts. In contrast, vaccination had a clear impact, enhancing circulating leukocyte numbers, particularly lymphocytes.

#### 3.4.2 Hepatic metabolites

Regardless of vaccination and dietary treatment, a transient increase in liver glycogen concentration was observed at 4 hpi, gradually decreasing at later time points (**Supplementary File S1**; **Supplementary Table S13**). In contrast, glucose levels significantly increased after infection and remained elevated throughout the post-infection period. TAG levels were increased at 24 and 48 hpi, and cholesterol levels peaked at 24 hpi. While lactate levels remained stable in vaccinated fish, a gradual decrease was observed in naïve fish, displaying significantly lower levels at 48 hpi compared to previous time points and to those observed in vaccinated fish at the same time point (**Supplementary File S1**; **Supplementary Table S13**).

#### 3.4.3 Oxidative stress markers

Hepatic CAT activity post-infection was affected by time and vaccination (**Supplementary File S1**; **Supplementary Table S14**). Naïve fish exhibited increased CAT activity following infection, whereas vaccinated fish experienced a transient decrease at 4 hpi, leading to significantly lower CAT activity at that time compared to naïve fish. On the opposite, SOD activity gradually decreased across the post-infection period in naïve fish, while in vaccinated fish a decline was observed only at 48 hpi. Even so, at 48 hpi, SOD activity was significantly higher in vaccinated fish compared to naïve. Additionally, dietary treatment also influenced SOD activity, with MET-fed fish exhibiting lower activity than CTRL-fed fish (**Supplementary File S1**; **Supplementary Table S14**).

For tGSH, no time-dependent changes were observed in CTRL-fed fish. However, in fish fed the MET diet, tGSH levels decreased at 48 hpi compared to earlier time points and were lower than those in CTRL-fed fish at that time point. Despite these results, no dietary effects were observed for rGSH or GSSG. At 4 hpi, vaccinated fish had lower rGSH levels than naïve fish, while GSSG levels decreased consistently across all groups post-infection. At 48 hpi, the GSH/GSSG ratio was increased in MET naïve fish compared to MET vaccinated fish. LPO levels were elevated in vaccinated fish at 4 hpi compared to naïve fish (**Supplementary File S1**; **Supplementary Table S14**).

In summary, vaccinated fish displayed lower CAT activity and rGSH levels, and higher LPO levels at 4 hpi, while SOD activity was higher in vaccinated than in naïve fish at 48 hpi. Dietary methionine supplementation also influenced antioxidant parameters, with MET-fed fish exhibiting lower SOD activity and tGSH levels at 48 hpi compared to CTRL-fed fish.

#### 3.4.4 Hepatic gene expression

Post-infection, mRNA *gdh* expression increased from 4 hpi onward in CTRL-fed fish, with no differences between naïve and vaccinated groups. In MET-fed fish, *gdh* levels peaked at distinct times depending on the vaccination status, with the highest expression observed at 24 hpi in naïve fish and at 48 hpi in vaccinated fish. The other metabolism-related gene studied, *hoad*, showed a marked increase at 48 hpi across all groups (**Supplementary File S1**; **Supplementary Table S15**).

Most genes associated with the methionine cycle were modulated in response to the bacterial challenge, with the exception of *sms* (**Supplementary File S1**; **Supplementary Table S15**). A transient increase in *sat1* expression was observed at 4 hpi, followed by a return to baseline levels at later time points. Expression of *bhmt* increased at 48 hpi, while *sahh1* levels decreased until 24 hpi before slightly rising at 48 hpi. Post-infection, *amd1* levels gradually increased until 24 hpi and then subtly declined by 48 hpi. Hepatic *amd1* expression was also influenced by dietary treatment and vaccination, with CTRL-fed fish displaying higher levels than MET-fed fish and vaccinated fish showing higher levels compared to naïve fish.

Regarding bacterial recognition receptors, *tlr1*, which recognizes bacterial lipoproteins, showed decreased expression following infection, whereas *tlr5*, responsible for detecting bacterial flagellin, increased, peaking at 24 hpi before returning to baseline levels by 48 hpi. Higher *tlr5* expression was observed in naïve fish compared to vaccinated fish. Regardless of treatment, a transient decrease in *leap2* expression occurred at 4 hpi, followed by a progressive increase through 48 hpi.

For adaptive immune-related genes, neither *cd8b* nor *migd* expression varied across the post-infection time points (**Supplementary File S1**; **Supplementary Table S15**).

In general, despite temporal fluctuations in gene expression following infection, *amd1* levels were higher in the liver of vaccinated and CTRL-fed fish compared to their respective naïve and MET-fed counterparts. Additionally, naïve fish exhibited higher *tlr5* expression than vaccinated fish.

#### 3.4.5 Gene expression in the head-kidney

Except for *il10*, all innate immune-related genes showed dynamic changes in the head-kidney during the post-infection period (**Supplementary File S1**; **Supplementary Table S16**). Expression levels of *saa* and *il1b* peaked at 24 hpi, with significantly higher *saa* levels observed in MET-fed fish. While *il8* expression remained stable in vaccinated fish, an increase over time was evident in naïve fish, reaching its highest levels at 24 hpi, where expression was significantly higher than in vaccinated fish. Similarly, *tnfa* expression peaked at 24 hpi in naïve fish, resulting in significantly higher levels compared to vaccinated fish at this time. Contrary to the observed in the liver (**Supplementary File S1**; **Supplementary Table S15**), *tlr1* expression levels in the head-kidney increased post-infection and were higher in naïve fish compared to vaccinated fish (**Supplementary File S1**; **Supplementary Table S16**).

Adaptive immune-related genes displayed distinct expression patterns. A gradual increase in *igm* expression was observed in all groups post-infection, while *migd* expression increased only at 24 and 48 hpi, and *igt* expression remained unchanged. Most cellular markers of adaptive immunity, such as *cd4*, *cd8a* and *mhci*, remained unchanged. However, *cd8b* expression was significantly higher in MET-fed fish compared to CTRL-fed fish at 48 hpi (**Supplementary File S1**; **Supplementary Table S16**).

Overall, naïve fish consistently exhibited higher *tlr1* expression, along with elevated *il8* and *tnfa* levels at 24 hpi, compared to their vaccinated counterparts. Furthermore, the MET diet was associated with increased *saa* expression and higher *cd8b* levels at 48 hpi.

#### 3.4.6 Principal component analysis

**Figure 5:**
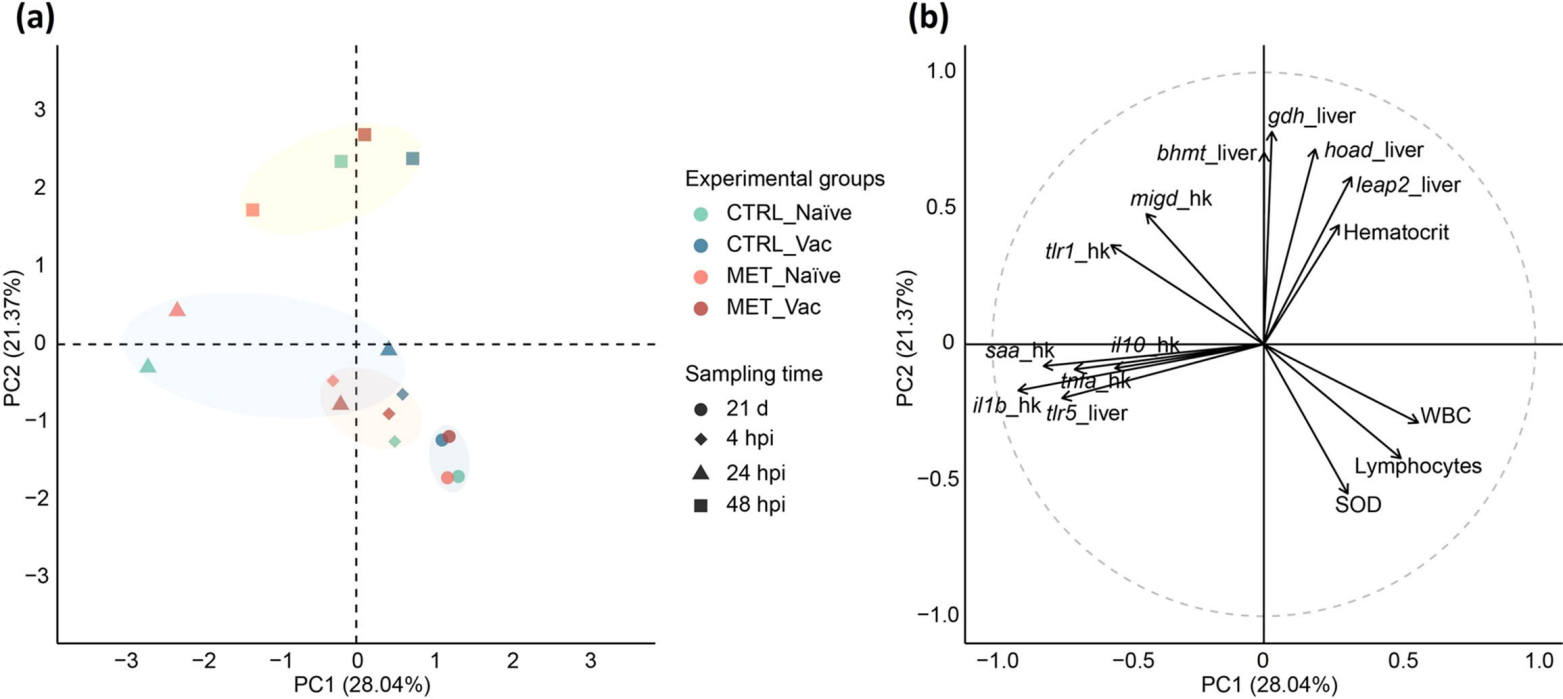
Principal component analysis integrating results from hematology, hepatic antioxidant capacity and gene expression analysis in the head-kidney and liver of rainbow trout fed different dietary treatments (CTRL or MET) for 7 weeks (4 weeks before vaccination and 3 weeks post-vaccination), evaluated at 21 days post-vaccination (21d), and at 4, 24 and 48 hpi. **(a)** PCA plot with centroid scores for each experimental group. **(b)** Correlation circle plot displaying the variables most strongly correlated with the first two principal components (PC1 and PC2).

All results were integrated into a PCA to provide a multivariate perspective and capture overall patterns and relationships among the variables influencing the immune response of rainbow trout to *Y. ruckeri*. The first two principal components (PCs) accounted for 49.41% of the total dataset variability (**Figure 5**; PC1 28.04% and PC2 21.37%).

Prior to the bacterial challenge, all experimental groups clustered together, primarily influenced by positive associations with hepatic SOD activity, circulating lymphocytes and total WBC numbers. By 4 hpi, the influence of these variables diminished, especially in MET naïve fish.

At 24 hpi, a clear distinction emerged between naïve and vaccinated fish. Naïve fish were positively associated with hepatic *tlr5* expression and the expression of *tlr1*, *il10*, *saa*, *il1b*, *tnfa* and *migd* in the head-kidney. In contrast, vaccinated groups clustered near the origin (0,0), suggesting weaker discrimination by the analyzed parameters.

By 48 hpi, MET naïve fish exhibited the greatest separation from the other groups. While CTRL naïve and, particularly, both vaccinated groups, were positively associated with hepatic *gdh*, *bhmt*, *hoad* and *leap2* expression, as well as hematocrit levels, MET naïve fish had a weaker association with these variables. Instead, this group remained more positively associated with the same inflammatory markers observed at 24 hpi (i.e., hepatic *tlr5* and *tlr1*, *il10*, *saa*, *il1b*, *tnfa* and *migd* in the head-kidney), as well as *tlr1* and *migd* expression in the head-kidney, and negatively associated with hepatic SOD, and circulating total WBC and lymphocytes counts.

### 3.5 Plasma proteomic analysis

**Figure 6:**
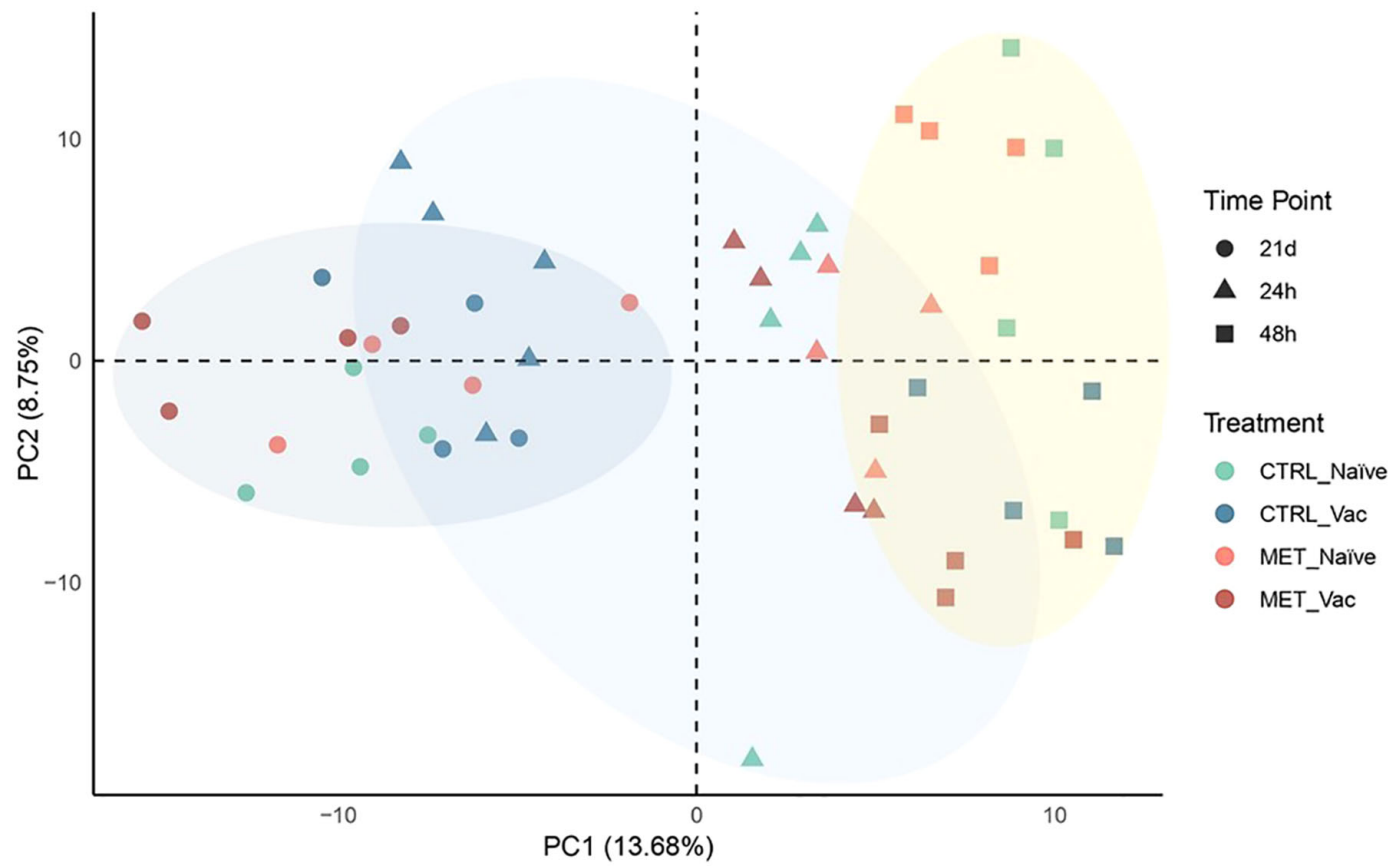
Principal component analysis showing the clustering of proteomic data from the plasma of rainbow trout fed experimental diets (CTRL, MET) for seven weeks (4 weeks before vaccination and 3 weeks post-vaccination) and sampled at 21 days post-vaccination against *Y. ruckeri* (21d), as well as at 24 and 48 hpi with the same pathogen.

Plasma samples collected after seven weeks (4 weeks before vaccination and 3 weeks post-vaccination) of feeding on experimental diets (21d), and at 24 and 48 hpi, were subjected to proteomic analysis. A total of 464 proteins were identified. The PCA revealed limited separation between the groups, with the first two PCs explaining 22.43% of the total variance in the dataset (**Figure 6**). Nevertheless, two main distinct clusters emerged: one corresponding to pre-infection samples and the other to post-infection samples. Interestingly, the CTRL vaccinated group at 24 hpi clustered more closely with the pre-infection groups than with the post-infection groups. Moreover, by 48 hpi, vaccinated groups were more tightly grouped and distinctly separated from the naïve fish along PC2.

#### 3.5.1 Differential abundance analysis

**Figure 7:**
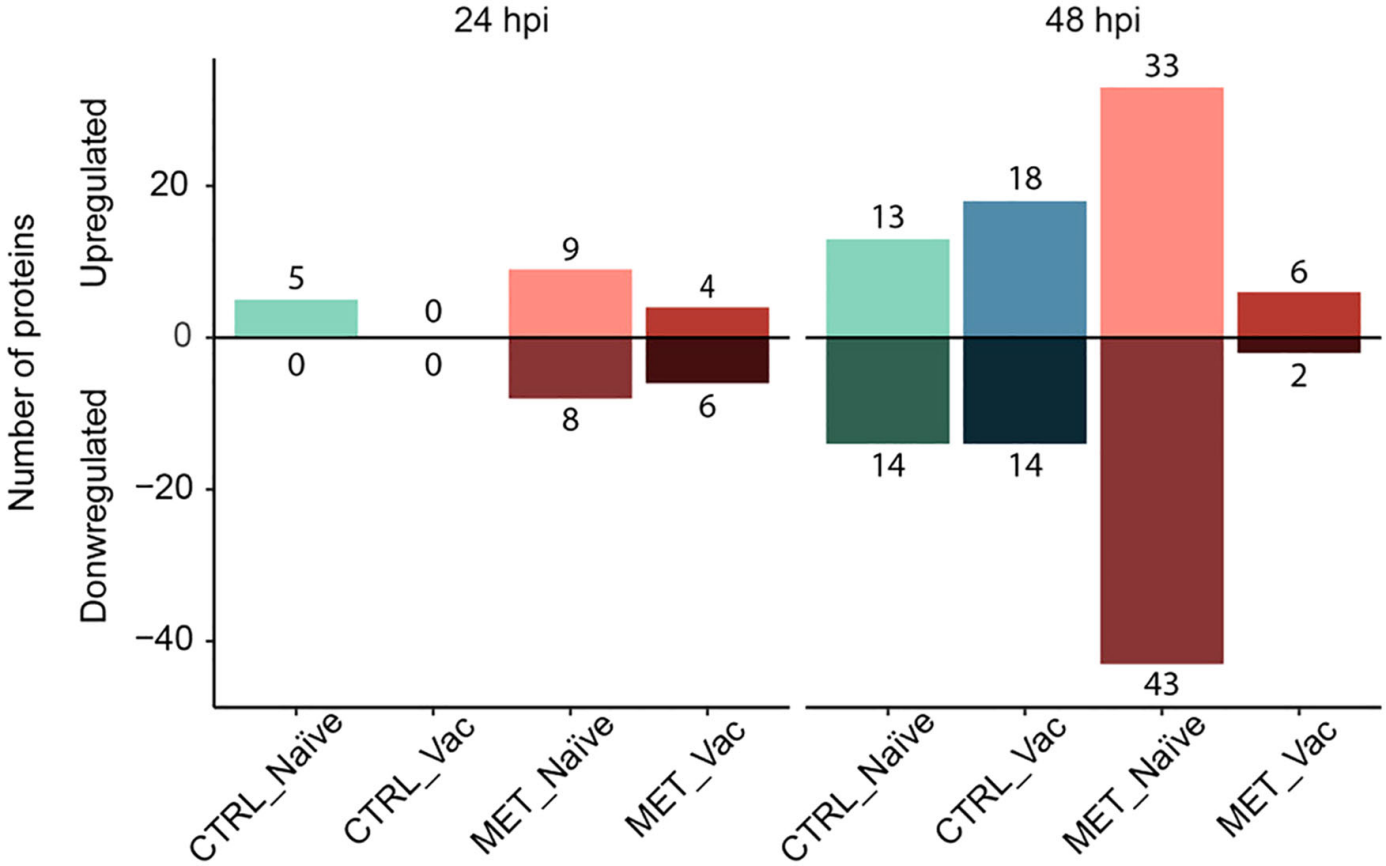
Diverted stacked bar chart showing differentially abundant proteins (*p*_adj_ < 0.05) in the plasma of rainbow trout fed experimental diets (CTRL, MET) for seven weeks (4 weeks before vaccination and 3 weeks post-vaccination against *Y. ruckeri*), at 24 and 48 h post-infection with the same pathogen.

Differential abundance analysis comparing all groups at 21d, 24 and 48 hpi against the CTRL naïve group at 21d identified a total of 90 DAPs (**Supplementary File S2**). While no DAPs were identified before infection, differences in the number of DAPs across groups were observed post-infection (**Figure 7**).

At 24 hpi, no DAPs were observed in the CTRL vaccinated fish, aligning with the clustering of this group with pre-infection groups in the PCA plot (**Figure 6**). The CTRL naïve group exhibited 5 upregulated DAPs, followed by the MET vaccinated group with 10 DAPs (4 upregulated, 6 downregulated) and the MET naïve group with 17 DAPs (9 upregulated, 8 downregulated) (**Figure 7**).

At 48 hpi, the MET naïve group again displayed the highest number of DAPs (76 total), with 33 upregulated and 43 downregulated. In contrast, the MET vaccinated group had the fewest (8 total), with 6 upregulated and 2 downregulated proteins. The CTRL-fed groups showed similar DAP counts, with the CTRL naïve group having 27 DAPs (13 upregulated and 14 downregulated) and the CTRL vaccinated group exhibiting 32 DAPs (18 upregulated and 14 downregulated).

**Figure 8:**
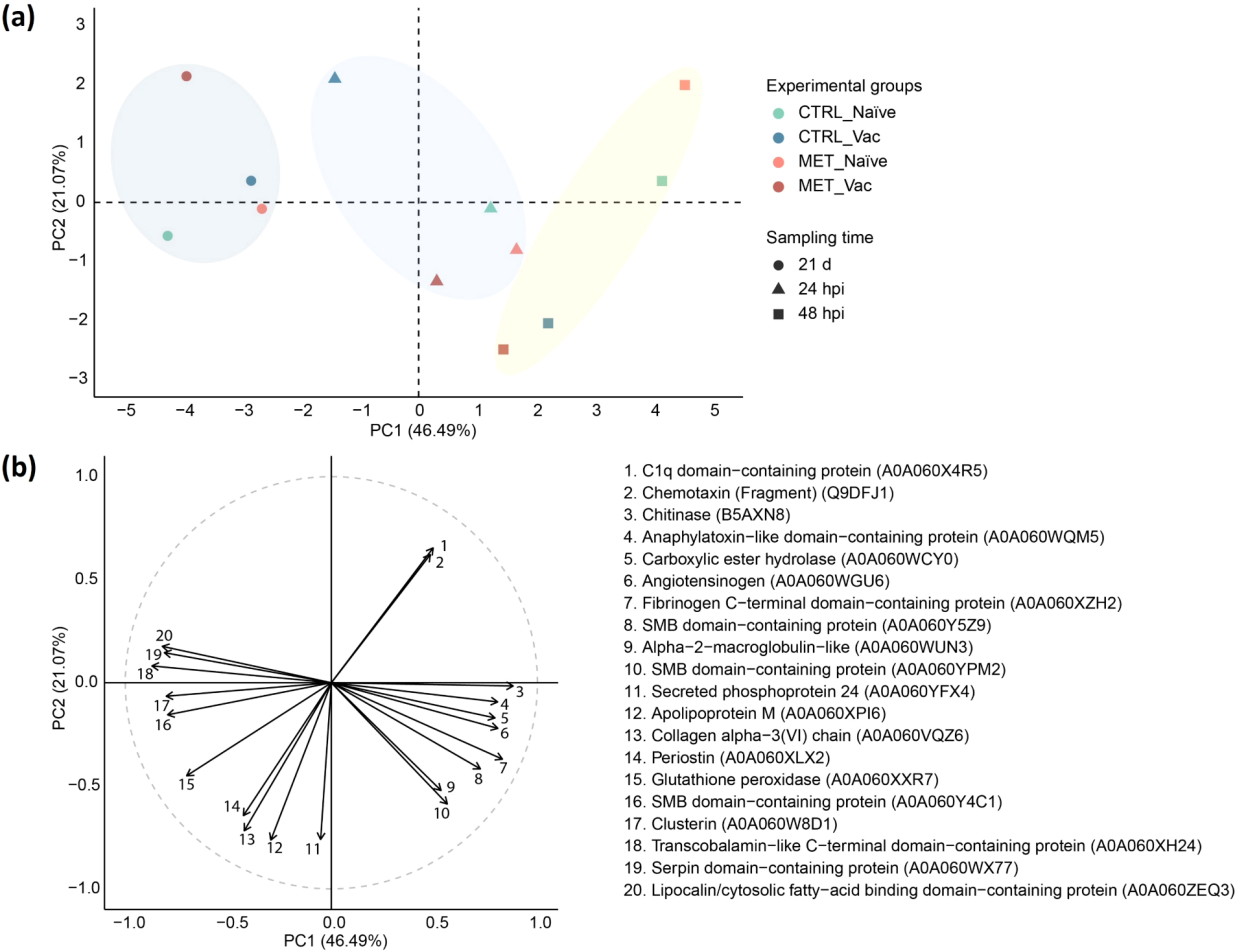
Principal component analysis (PCA) of differentially abundant plasma proteins (*p*_adj_ < 0.05) in rainbow trout fed experimental diets (CTRL, MET) for seven weeks (4 weeks before vaccination and 3 weeks post-vaccination against *Y. ruckeri*), at 24 and 48 hpi with the same pathogen. **(a)** PCA score plot showing group centroids. **(b)** Correlation circle plot displaying the top 20 proteins most strongly correlated with the principal components.

A PCA was conducted to assess the contribution of DAPs to the separation of experimental groups. A total of 67.56% of the variability of the dataset was explained by the first two PCs, with PC1 accounting for 46.49% and PC2 for 21.07% (**Figure 8**).

Before infection (21d), all groups were positively associated with lipocalin/cytosolic fatty-acid binding domain-containing protein (A0A060ZEQ3), serpin domain-containing protein (A0A060WX77), transcobalamin-like C-terminal domain-containing protein (A0A060XH24), clusterin (A0A060W8D1) and somatomedin B (SMB) domain-containing protein (A0A060Y4C1) (**Figure 8**). Among them, MET vaccinated and CTRL naïve showed the strongest associations with these variables. However, MET vaccinated fish were more negatively associated with alpha-2-macroglobulin-like (A0A060WUN3), SMB domain-containing protein (A0A060YPM2), apolipoprotein M (A0A060XPI6) and secreted phosphoprotein 24 (A0A060YFX4), whereas CTRL naïve fish displayed stronger positive association with periostin (A0A060XLX2), collagen alpha−3(VI) chain (A0A060VQZ6), apolipoprotein M (A0A060XPI6) and secreted phosphoprotein 24 (A0A060YFX4). Despite these differences, no DAPs were identified before infection.

By 24 hpi, all groups except CTRL vaccinated fish were positively associated with chitinase (B5AXN8), anaphylatoxin-like domain-containing protein (A0A060WQM5), carboxylic ester hydrolase (A0A060WCY0), angiotensinogen (A0A060WGU6), fibrinogen C-terminal domain-containing protein (A0A060XZH2) and SMB domain-containing protein (A0A060Y5Z9). Additionally, MET-fed groups were also positively loaded by alpha-2-macroglobulin-like (A0A060WUN3) and SMB domain-containing protein (A0A060YPM2). In contrast, CTRL vaccinated fish showed a distinct pattern, being more positively associated with C1q domain-containing protein (A0A060X4R5) and chemotaxin (Fragment) (Q9DFJ1) and negatively with alpha-2-macroglobulin-like (A0A060WUN3), SMB domain-containing protein (A0A060YPM2), periostin (A0A060XLX2), collagen alpha-3(VI) chain (A0A060VQZ6), apolipoprotein M (A0A060XPI6) and secreted phosphoprotein 24 (A0A060YFX4) (**Figure 7**). Nevertheless, no DAPs were identified in this group at 24 hpi (**Figure 7**).

At 48 hpi, both naïve groups were more strongly associated with chitinase (B5AXN8), anaphylatoxin-like domain-containing protein (A0A060WQM5) and carboxylic ester hydrolase (A0A060WCY0), while being more negatively associated with clusterin (A0A060W8D1), transcobalamin-like C-terminal domain-containing protein (A0A060XH24) and SMB domain-containing protein (A0A060Y4C1) compared to their vaccinated counterparts. Among the naïve groups, MET fed fish were most influenced by C1q domain-containing protein (A0A060X4R5) and chemotaxin (Fragment) (Q9DFJ1), while negatively associated with glutathione peroxidase (A0A060XXR7) and periostin (A0A060XLX2). In contrast, both vaccinated groups were positively associated with periostin (A0A060XLX2), collagen alpha-3(VI) chain (A0A060VQZ6), apolipoprotein M (A0A060XPI6), secreted phosphoprotein 24 (A0A060YFX4), alpha-2-macroglobulin-like (A0A060WUN3) and SMB domain-containing protein (A0A060YPM2) (**Figure 8**).

Given the focus of this study on the effects of dietary methionine supplementation, it is important to highlight that the metabolic proteins 6-phosphogluconate dehydrogenase (A0A060WX43) and triosephosphate isomerase (A0A060VUA9) were exclusively significantly upregulated in MET-fed naïve fish at 48 hpi (**Supplementary File S2**).

## 4 Discussion

### 4.1 Immune status after feeding with methionine

Regardless of vaccination status, seven weeks of methionine supplementation had minimal effects on the rainbow trout immune status, primarily reducing hepatic *amd1* expression. This finding aligns with previous studies (13) and suggests inhibition of the aminopropylation pathway and downstream polyamine biosynthesis (47). Polyamines (i.e., putrescine, spermine and spermidine) are essential for cellular processes, including proliferation, differentiation, apoptosis and autophagy (48). They are also recognized for their antioxidant and anti-inflammatory properties (49–51). Given these crucial roles, the decrease in *amd1* expression could potentially be related to fish health. However, no additional effects of dietary methionine supplementation on the fish immune status were observed.

Similarly, at 21 days post-vaccination, differences between the immune status of vaccinated and naïve fish were minor. Regardless of dietary treatment, vaccinated fish displayed elevated MCV levels compared to their naïve counterparts. However, this effect was likely transient and of limited physiological significance, as it was absent at all post-infection time points and the other hematological parameters were comparable between the two groups. Additionally, vaccinated fish exhibited reduced *cd8b* expression in the head-kidney, along with a trend toward lower *cd8a*. This observation is consistent with studies in Atlantic salmon (*Salmo salar*) vaccinated against *Piscirickettsia salmonis*, where downregulation of *cd8b* in the head-kidney suggested a promotion of T CD4^+^ cell responses (52). However, unchanged *cd4* expression in the present study points to alternative vaccination-induced mechanisms.

Despite these gene expression differences, plasma proteomic analysis revealed that, prior to infection, all experimental groups closely resembled the CTRL naïve fish, with no detectable DAPs.

### 4.2 Primary and secondary immune responses to *Yersinia ruckeri* infection

In the present study, *Y. ruckeri* infection elicited common immune responses across all experimental groups, including a decrease in circulating total WBC, driven by a reduction in lymphocyte numbers, and increased expression of pro-inflammatory genes in the head-kidney, similar to that observed previously (53).

Despite these shared responses to infection, as anticipated, the immune response greatly differed between naïve and vaccinated fish (54–57). For instance, naïve fish appeared to rely on TLR5 for bacterial recognition regardless of dietary treatment, as indicated by the increased hepatic *tlr5* mRNA expression at 24 hpi compared to the vaccinated counterparts. Previous studies have demonstrated that *Y. ruckeri* flagellin binds to TLR5, triggering cytokine transcription in the liver of rainbow trout during their first exposure to the pathogen (56). Following bacterial recognition, the expression of the pro-inflammatory cytokines *tnfa* and *il8* was markedly increased exclusively in the head-kidney of naïve fish (58, 59), highlighting their importance in the early primary immune response. While TNFα plays a central role in driving inflammation (60), IL8 is primarily involved in the activation and recruitment of neutrophils to the site of infection (61).

In the present study, the early secondary immune response in vaccinated fish was characterized by increased hepatic LPO levels irrespectively of dietary treatment, alongside decreased CAT activity and rGSH levels, indicative of oxidative stress. This oxidative burst likely reflects a faster and more robust activation of phagocytes or enhanced respiratory burst activity in vaccinated fish (58, 62). The rapid resolution of infection in vaccinated fish, evidenced by the minimal bacterial loads and the absence of detectable bacteria from 24 hpi onward, support this hypothesis. Additionally, vaccinated fish exhibited lower expression levels of innate immune-related genes, including *il8*, *tnfa* and *tlr1*, in the head-kidney compared to their naïve counterparts. These findings align with previous studies reporting a rapid release of ROS upon immune activation (58, 62), a prompt resolution of infection within the first hours post-challenge, with no detectable bacteria by one day post-infection (55) and minimal pro-inflammatory gene expression in *Y. ruckeri*-vaccinated fish (55, 57). Furthermore, in the present study, by 24 hpi both CAT activity and LPO levels in vaccinated fish had returned to baseline levels, and by 48 hpi, SOD activity was significantly higher compared to naïve fish, indicating a sustained antioxidant response.

These distinct immune responses likely accounted for the higher bacterial loads, greater number of fish with detectable bacteria and, ultimately, lower survival rates observed in naïve fish compared to the vaccinated fish.

### 4.3 The modulatory role of methionine intake after infection

Focusing on the specific effects of dietary methionine supplementation on the primary and secondary immune responses of rainbow trout to *Y. ruckeri* infection, the present study revealed that, while hepatic *amd1* expression was upregulated in response to infection across all groups, MET-fed fish and naïve fish exhibited lower expression levels compared to CTRL-fed fish and vaccinated fish, respectively. This pattern was consistent with that observed before infection and suggests a potential impairment in polyamine biosynthesis. As previously mentioned, polyamines are absolutely essential for cellular viability and proliferation (48). Therefore, a reduction in *amd1* levels may hinder cell migration (63), potentially exacerbating inflammation and delaying recovery. This could partly be linked to the increased serum amyloid A (*saa*) expression observed in the head-kidney of MET-fed fish, particularly MET naïve fish. As an acute-phase protein, elevated *saa* expression is indicative of ongoing inflammation (64). The sustained upregulation of this gene in MET naïve fish at 48 hpi likely reflects persistent infection, which aligns with the exclusive presence of bacteria in this group at that time point. Furthermore, the concurrent, albeit not statistically significant, upregulation of *il10*, an anti-inflammatory cytokine (55), in this group, suggests a potential attempt to counterbalance inflammation and minimize potential tissue damage.

Consistent with the gene expression outcomes, MET naïve fish displayed increased abundance of several immune-related proteins in the plasma compared to CTRL naïve fish at 48 hpi. Notably, MET naïve fish had higher levels of C1q domain-containing protein, a key initiator of the classical complement pathway (65), and chemotaxin, a protein that mediates immune cell migration toward infection or injury sites to promote pathogen clearance (66). Simultaneously, MET naïve fish exhibited reduced levels of periostin, a protein associated with wound healing and tissue repair (67, 68). This prolonged immune activation in MET naïve fish compared to the other groups, along with the delayed resolution phase, may reflect a less efficient immune response, which potentially contributed to the higher bacterial loads and, ultimately, the lower survival rate recorded in this group.

Beyond these immune-related changes, MET naïve fish were the only to present significant alterations in metabolism-related proteins. The increased abundance of 6-phosphogluconate dehydrogenase in the plasma at 48 hpi suggests an activation of the pentose phosphate pathway (PPP), which generates reducing power in the form of reduced nicotinamide-adenine dinucleotide phosphate (NADPH) to support antioxidant defenses during oxidative stress (69). Additionally, MET naïve fish displayed increased abundance of triosephosphate isomerase, a central enzyme in carbohydrate metabolism and energy production, with its roles extending beyond glycolysis to include gluconeogenesis, PPP and fatty acid biosynthesis (70). These metabolic shifts likely reflect increased demand for NADPH and metabolic intermediates to sustain both antioxidant defense and immune function.

Additionally, both MET-fed groups displayed reduced SOD activity compared to CTRL-fed fish. Given that methionine is a precursor of cysteine, whose availability increases during injury via the transsulfuration pathway (71), we hypothesized that MET-fed fish could have relied more on glutathione (18) rather than on SOD for ROS detoxification. Consequently, higher tGSH and rGSH levels were expected in MET-fed fish. However, no statistically significant differences in hepatic rGSH were observed at any time point, and tGSH levels were significantly lower in MET-fed fish at 48 hpi compared to the CTRL group. Plasma proteomic analysis further revealed a reduction in glutathione peroxidase (GPx) abundance in MET naïve fish at 48 hpi. Since GPx neutralizes ROS by using GSH as a substrate, leading to GSSG generation, the decrease in GPx may have contributed to the lower GSSG levels and, consequently, higher GSH: GSSG ratio observed in this group at 48 hpi. Although this ratio is often associated with better redox balance (72), in this case it may reflect reduced GSH turnover or impaired antioxidant enzyme activity, rather than enhanced antioxidant capacity. Moreover, while LPO levels remained unchanged, oxidative damage is not limited to lipids. Therefore, it cannot be excluded that other molecules, such as proteins, may have been affected by ROS (73).

Taken together, these results suggest that, under the present conditions, the level of methionine supplementation tested (17.8 mg/g DW) may have exceeded the optimal threshold for enhancing fish immune function and antioxidant defenses. Consistent with these findings, a number of studies in fish reported that beyond a specific intake level, methionine supplementation yields no additional benefits and can even lead to a decline in immune and antioxidant defense mechanisms (17, 20, 24, 25, 74). Although the methionine supplementation level used in this study was well above the established requirement range for rainbow trout (0.55–0.75% of diet, or 1.57–2.14% of dietary protein) (75), it was selected based on previous studies reporting immune-enhancing effects in this species (14). Discrepancies among studies may arise from variations in fish size and batch, feeding duration, rearing conditions, basal diet composition and dietary cysteine levels, among other factors. Nevertheless, supporting our findings, Martin et al. (20) reported that a dietary methionine concentration of 15.8 mg/g DW could already surpass the optimal threshold for improving certain immune parameters in rainbow trout.

Due to methionine’s participation in several pathways and mechanisms capable of influencing immune function, pinpointing a single cause for the observed effects is challenging. Nevertheless, some authors attribute these negative effects to the accumulation of specific metabolites arising from the overloading of metabolic pathways, such as homocysteine (76, 77), or changes in DNA methylation patterns due to the higher availability of methyl donors (78).

Given the pivotal role of methionine in key immune processes, such as protein synthesis, cell differentiation and proliferation, and epigenetic regulation (19), and supported by previous studies demonstrating that adequate methionine supplementation increases the number of circulating leukocytes (11) and IgM-secreting cells, as well as IgM-secreting capacity of B cells (20), the present study hypothesized that dietary methionine supplementation could enhance vaccine efficacy. However, contrary to this initial hypothesis, no significant differences were observed between CTRL and MET vaccinated fish. This lack of clear effects may result from the relatively low challenge-induced mortality, together with the full protection observed in both vaccinated groups, which could have masked or diminished potential impacts of the dietary intervention. The most pronounced differences between the two groups emerged in the plasma proteome, particularly at 24 hpi. At this time, MET vaccinated fish showed an increase in hemostasis-related proteins (e.g., angiotensinogen, fibrinogen C-terminal domain-containing protein and alpha-2-macroglobulin-like), whereas CTRL vaccinated fish displayed a response more akin to pre-infection groups. Hemostasis, which involves the rapid formation of fibrin clots to prevent blood loss (79), is of utmost importance, especially during hemorrhagic diseases like ERM (80). In addition to preventing fluid loss, clot formation contributes to immune defense by physically trapping bacteria, preventing their dissemination (81). It also enhances vascular permeability, acts as a chemotactic signal for phagocytes and activates the complement cascade (82), a key component of innate and adaptive immunity (83). Moreover, beyond its structural role in clot stabilization, fibrinogen also possesses antimicrobial properties (84). In this study, fibrinogen C-terminal domain-containing protein abundance was increased in the plasma of all groups at 24 hpi, except in the CTRL vaccinated group, where its increase was delayed until 48 hpi. This temporal difference may be linked to the persistence of bacteria in CTRL vaccinated fish at 24 hpi, which was not observed in the MET vaccinated group. Although this suggests that methionine supplementation could facilitate earlier bacterial clearance when combined with vaccination, it is important to acknowledge the limitations of the bacterial quantification method used, which prevented detection of bacteria at concentrations below 230 CFU/mL. This limitation, along with other factors such as tissue damage caused by the bacterium or a heightened pro-inflammatory response, may also help explain why bacterial clearance in naïve fish appeared nearly complete by 48 hpi, despite mortality only beginning on day 3 post-infection.

By 48 hpi, the proteomic profiles of both vaccinated groups converged, marked by a shared positive association with proteins related to tissue remodeling and wound healing (e.g., secreted phosphoprotein 24, apolipoprotein M, collagen alpha-3(VI) chain and periostin). Moreover, no mortality was observed in either vaccinated group, further supporting that dietary methionine supplementation, at the tested level, had minimal effects on improving the efficacy of the *Y. ruckeri* vaccine under the present experimental conditions. This outcome may also reflect the relatively low inoculum dose of *Y. ruckeri* used in the experiment, which could have limited the magnitude of the immune response in vaccinated fish. Vaccine efficacy is typically assessed using a challenge at LD70 or higher. The rapid pathogen clearance observed in immunized rainbow trout likely masked any potential effect of dietary methionine supplementation.

Nevertheless, vaccination appeared to positively influence the immune response of MET-fed fish. This improvement may be attributed to specific biological mechanisms triggered by the vaccine itself. For instance, while MET-fed fish exhibited reduced hepatic *amd1* expression compared to CTRL-fed fish, vaccinated fish, regardless of dietary treatment, displayed elevated *amd1* levels compared to their naïve counterparts. This suggests that vaccination may enhance polyamine biosynthesis through mechanisms independent of methionine intake. By promoting polyamine production, vaccination likely facilitated tissue repair and immune resolution in MET vaccinated fish (63). Similarly, although MET-fed fish displayed reduced SOD activity, vaccination resulted in a significant increase in the enzyme activity, indicating that the vaccine may have supported antioxidant defenses, compensating for the negative impact of methionine supplementation on SOD activity.

## 5 Conclusion

In conclusion, naïve rainbow trout infected with *Y. ruckeri* exhibited a higher bacterial prevalence, lower survival rates, increased pro-inflammatory gene expression and a greater abundance of immune-related proteins. In contrast, vaccinated fish seemed to resolve the infection more rapidly, possibly favored by an early and heightened production of ROS.

Dietary methionine supplementation at the tested level (17.8 mg methionine/g DW) appeared to impair antioxidant defenses and prolong immune activation in naïve rainbow trout, potentially due to a less efficient response, which may have contributed to their lower survival rate. Therefore, these data suggest that dietary methionine intake exceeded a safe threshold in non-immunized fish, yet did not seem to impair the response to the vaccine.

Differences between the two vaccinated groups were subtle. Yet, the variations in the plasma proteome observed at 24 hpi may be partly related to the slightly delayed pathogen clearance in CTRL vaccinated fish compared to MET vaccinated fish. Nevertheless, no mortality was observed in either vaccinated group, and whether the faster bacterial clearance observed in MET vaccinated fish reflects a true beneficial effect of methionine supplementation in combination with vaccination or not, remains to be determined. Further studies under more challenging infection conditions, together with the assessment of *Y. ruckeri*-specific antibody production, are required to confirm this potential synergistic interaction and to better characterize the adaptive immune response induced by this combined strategy.

## Data Availability

The datasets presented in this study can be found in online repositories. The names of the repository/repositories and accession number(s) can be found below: https://www.ebi.ac.uk/pride/archive/projects/PXD068409, PXD068409 https://doi.org/10.6084/m9.figshare.30138397.v1, 10.6084/m9.figshare.30138397.
