# Supplementary material for: Immunomodulatory effects of dietary methionine supplementation in rainbow trout (Oncorhynchus mykiss) juveniles: insights following vaccination and infection response against Yersinia ruckeri
Source: Front Immunol. 2025 Nov 3;16:1706922. doi: 10.3389/fimmu.2025.1706922 (PMC12620199; doi:10.3389/fimmu.2025.1706922)
Supplement: Supplementary file 1 [file Table1.docx]

***Supplementary Material***

**Table S1.** Hematocrit, hemoglobin, mean corpuscular volume (MCV), mean corpuscular hemoglobin (MCH), mean corpuscular hemoglobin concentration (MCHC), red blood cells (RBC) and white blood cells (WBC) in rainbow trout fed dietary treatments for 7 weeks (4 weeks before vaccination and 3 weeks post-vaccination). Values are presented as mean ± SD (n = 8). “x” and “y” stand for differences between naïve and vaccinated fish (two-way ANOVA; *p* ≤ 0.05).

| **Parameters** | | | **CTRL** | | | | **MET** | | | |
| --- | --- | --- | --- | --- | --- | --- | --- | --- | --- | --- |
|  |  |  | **Naïve** | | **Vac** | | **Naïve** | | **Vac** | |
| **Hematocrit** | **(%)** | | 44.75 ± 3.69 | | 45.13 ± 3.56 | | 42.88 ± 3.64 | | 46.13 ± 4.02 | |
| **Hemoglobin** | **(g/dL)** | | 1.28 ± 0.36 | | 1.46 ± 0.20 | | 1.52 ± 0.08 | | 1.44 ± 0.26 | |
| **MCV** | **(µm^3^)** | | 596.28 ± 143.54 | | 662.02 ± 140.96 | | 534.35 ± 67.67 | | 709.63 ± 203.54 | |
| **MCH** | **(pg/cell)** | | 18.73 ± 4.20 | | 21.64 ± 5.86 | | 19.78 ± 5.87 | | 24.10 ± 7.44 | |
| **MCHC** | **(g/100 mL)** | | 2.86 ± 0.73 | | 3.25 ± 0.40 | | 3.37 ± 0.50 | | 3.11 ± 0.44 | |
| **RBC** | **(× 10^6^/µL)** | | 0.80 ± 0.25 | | 0.70 ± 0.15 | | 0.76 ± 0.19 | | 0.68 ± 0.16 | |
| **WBC** | **(× 10^4^/µL)** | | 4.79 ± 1.46 | | 4.59 ± 0.86 | | 4.44 ± 0.78 | | 4.19 ± 0.74 | |
|  | |  | |  | |  | | **Vaccination** | | |
|  | | **Diet** | | **Vaccination** | | **Diet × vaccination** | | **Naïve** | | **Vac** |
| **Hematocrit** | | ns | | ns | | ns | | - | | - |
| **Hemoglobin** | | ns | | ns | | ns | | - | | - |
| **MCV** | | ns | | 0.026 | | ns | | y | | x |
| **MCH** | | ns | | ns | | ns | | - | | - |
| **MCHC** | | ns | | ns | | ns | | - | | - |
| **RBC** | | ns | | ns | | ns | | - | | - |
| **WBC** | | ns | | ns | | ns | | - | | - |

**Table S2.** Absolute values of peripheral blood leucocytes (thrombocytes, lymphocytes, monocytes and neutrophils) in rainbow trout rainbow trout fed dietary treatments for 7 weeks (4 weeks before vaccination and 3 weeks post-vaccination). Values are presented as means ± SD (n = 8). *P*-values from two-way ANOVA; *p* ≤ 0.05.

| **Parameters** | | | **CTRL** | | | **MET** | | |
| --- | --- | --- | --- | --- | --- | --- | --- | --- |
|  |  |  | **Naïve** | | **Vac** | **Naïve** | | **Vac** |
| **Thrombocytes** | | **(×10^4^/µL)** | 0.74 ± 0.36 | | 0.69 ±0.35 | 0.82 ± 0.62 | | 0.36 ± 0.06 |
| **Lymphocytes** | |  | 3.75 ± 1.59 | | 3.55 ± 0.59 | 3.28 ± 0.70 | | 3.49 ± 0.80 |
| **Monocytes** | |  | 0.08 ± 0.06 | | 0.10 ± 0.05 | 0.11 ± 0.13 | | 0.11 ± 0.06 |
| **Neutrophils** | |  | 0.21 ± 0.18 | | 0.30 ± 0.20 | 0.22 ± 0.21 | | 0.24 ± 0.09 |
|  | **Diet** | | | **Vaccination** | | | **Diet × vaccination** | |
| **Thrombocytes** | ns | | | ns | | | ns | |
| **Lymphocytes** | ns | | | ns | | | ns | |
| **Monocytes** | ns | | | ns | | | ns | |
| **Neutrophils** | ns | | | ns | | | ns | |

**Table S3.** Triglycerides (TAG), cholesterol, glucose, glycogen and lactate levels in the liver of rainbow trout fed dietary treatments for 7 weeks (4 weeks before vaccination and 3 weeks post-vaccination). Values are presented as means ± SD (n = 8). *P*-values from two-way ANOVA; *p* ≤ 0.05.

| **Parameters** | | **CTRL** | | | | **MET** | | |
| --- | --- | --- | --- | --- | --- | --- | --- | --- |
|  |  | **Naïve** | | **Vac** | | **Naïve** | | **Vac** |
| **TAG** | **(mg/g ww)** | 21.44 ± 1.60 | | 18.92 ± 4.83 | | 20.58 ± 1.55 | | 21.92 ± 5.53 |
| **Cholesterol** |  | 0.46 ± 0.13 | | 0.44 ± 0.05 | | 0.44 ± 0.04 | | 0.46 ± 0.09 |
| **Glucose** |  | 45.45 ± 14.84 | | 38.31 ± 16.60 | | 39.95 ± 16.19 | | 35.77 ± 14.47 |
| **Glycogen** |  | 144.19 ± 79.22 | | 231.82 ± 82.75 | | 167.98 ± 108.89 | | 195.00 ± 113.33 |
| **Lactate** |  | 0.25 ± 0.05 | | 0.20 ± 0.09 | | 0.25 ± 0.04 | | 0.23 ± 0.07 |
|  | | | **Diet** | | **Vaccination** | | **Diet × vaccination** | |
| **TAG** | | | ns | | ns | | ns | |
| **Cholesterol** | | | ns | | ns | | ns | |
| **Glucose** | | | ns | | ns | | ns | |
| **Glycogen** | | | ns | | ns | | ns | |
| **Lactate** | | | ns | | ns | | ns | |

**Table S4.** Catalase activity (CAT), superoxide dismutase (SOD), total glutathione (tGSH), reduced glutathione (rGSH), oxidized glutathione (GSSG) and lipid peroxidation levels (LPO) in the liver of rainbow trout fed dietary treatments for 7 weeks (4 weeks before vaccination and 3 weeks post-vaccination). Values are presented as means ± SD (n = 8). *P*-values from two-way ANOVA; *p* ≤ 0.05.

| **Parameters** | | | **CTRL** | | | **MET** | | |
| --- | --- | --- | --- | --- | --- | --- | --- | --- |
|  |  |  | **Naïve** | | **Vac** | **Naïve** | | **Vac** |
| **CAT** | **(units/mg protein)** | | 161.52 ± 43.76 | | 211.01 ± 39.23 | 156.93 ± 30.72 | | 161.55 ± 60.02 |
| **SOD** | **(units/mg protein)** | | 95.58 ± 9.78 | | 78.79 ± 15.76 | 92.78 ± 17.99 | | 86.98 ± 9.55 |
| **tGSH** | **(µM)** | | 1246.72 ± 363.38 | | 1324.06 ± 183.06 | 1377.75 ± 220.90 | | 1368.05 ± 313.32 |
| **rGSH** | **(µM)** | | 849.44 ± 317.94 | | 977.39 ± 250.56 | 980.69 ± 201.19 | | 992.78 ± 220.70 |
| **GSSG** | **(µM)** | | 198.64 ± 51.24 | | 173.33 ± 79.73 | 198.73 ± 33.07 | | 187.64 ± 59.02 |
| **GSH/GSSG** |  | | 4.41 ± 1.96 | | 4.84 ± 1.36 | 5.06 ± 1.40 | | 5.63 ± 1.48 |
| **LPO** | **(nmol/g wt)** | | 43.46 ± 5.80 | | 42.34 ± 1.72 | 41.60 ± 4.48 | | 42.47 ± 4.34 |
|  | | **Diet** | | **Vaccination** | | | **Diet × vaccination** | |
| **CAT** | | ns | | ns | | | ns | |
| **SOD** | | ns | | ns | | | ns | |
| **tGSH** | | ns | | ns | | | ns | |
| **rGSH** | | ns | | ns | | | ns | |
| **GSSG** | | ns | | ns | | | ns | |
| **GSH/GSSG** | | ns | | ns | | | ns | |
| **LPO** | | ns | | ns | | | ns | |

**Table S5.** Quantitative expression of *bhmt*, *cd8b*, *gdh*, *hoad*, *leap2*, *migd*, *sahh1*, *amd1*, *sat1*, *sms*, *tlr1* and *tlr5* in the liver of rainbow trout fed dietary treatments for 7 weeks (4 weeks before vaccination and 3 weeks post-vaccination). Different capital letters stand for differences between dietary treatments. Values are presented as means ± SD (n = 8). *P*-values from two-way ANOVA; *p* ≤ 0.05.

| **Genes** | **CTRL** | | | | **MET** | | | | |
| --- | --- | --- | --- | --- | --- | --- | --- | --- | --- |
|  | **Naïve** | | **Vac** | | **Naïve** | | **Vac** | | |
| ***bhmt*** | 1.31 ± 1.11 | | 1.67 ± 2.39 | | 1.28 ± 0.62 | | 0.67 ± 0.36 | | |
| ***cd8b*** | 1.16 ± 0.76 | | 1.20 ± 1.15 | | 1.13 ± 0.87 | | 1.14 ± 1.10 | | |
| ***gdh*** | 1.17 ± 0.63 | | 0.83 ± 0.49 | | 0.78 ± 0.40 | | 1.19 ± 0.59 | | |
| ***hoad*** | 1.02 ± 0.26 | | 1.11 ± 0.38 | | 1.07 ± 0.27 | | 1.24 ± 0.39 | | |
| ***leap2*** | 1.12 ± 0.61 | | 1.29 ± 0.4 | | 1.08 ± 0.31 | | 1.35 ± 0.58 | | |
| ***migd*** | 1.10 ± 0.51 | | 1.19 ± 0.61 | | 0.76 ± 0.21 | | 1.15 ± 0.53 | | |
| ***sahh1*** | 1.24 ± 0.81 | | 1.49 ± 1.35 | | 1.22 ± 1.15 | | 1.25 ± 1.13 | | |
| ***amd1*** | 1.32 ± 0.89 | | 1.70 ± 0.73 | | 1.00 ± 0.39 | | 0.99 ± 0.49 | | |
| ***sat1*** | 1.11 ± 0.67 | | 1.03 ± 0.45 | | 0.98 ± 0.59 | | 1.42 ± 0.41 | | |
| ***sms*** | 1.10 ± 0.49 | | 1.66 ± 0.71 | | 1.15 ± 0.58 | | 1.20 ± 0.49 | | |
| ***tlr1*** | 1.22 ± 0.77 | | 1.14 ± 0.66 | | 0.89 ± 0.49 | | 0.94 ± 0.33 | | |
| ***tlr5*** | 1.36 ± 1.05 | | 0.96 ± 1.58 | | 0.86 ± 1.04 | | 1.78 ± 2.13 | | |
|  | |  | |  | |  | | **Diet** | |
|  | | **Diet** | | **Vaccination** | | **Diet × vaccination** | | **CTRL** | **MET** |
| ***bhmt*** | | ns | | ns | | ns | | - | - |
| ***cd8b*** | | ns | | ns | | ns | | - | - |
| ***gdh*** | | ns | | ns | | ns | | - | - |
| ***hoad*** | | ns | | ns | | ns | | - | - |
| ***leap2*** | | ns | | ns | | ns | | - | - |
| ***migd*** | | ns | | ns | | ns | | - | - |
| ***sahh1*** | | ns | | ns | | ns | | - | - |
| ***amd1*** | | 0.039 | | ns | | ns | | A | B |
| ***sat1*** | | ns | | ns | | ns | | - | - |
| ***sms*** | | ns | | ns | | ns | | - | - |
| ***tlr1*** | | ns | | ns | | ns | | - | - |
| ***tlr5*** | | ns | | ns | | ns | | - | - |

**Table S6.** Quantitative expression of *cd4*, *cd8a*, *cd8b*, *igm*, *igt*, *il1b*, *il8*, *il10*, *mhci*, *migd*, *tlr1*, *saa* and *tnfa* in the head kidney of rainbow trout fed dietary treatments for 7 weeks (4 weeks before vaccination and 3 weeks post-vaccination). Values are presented as means ± SD (n = 8). “x” and “y” stand for differences between naïve and vaccinated fish. *P*-values from two-way ANOVA; *p* ≤ 0.05.

| **Genes** | **CTRL** | | | | **MET** | | | | |
| --- | --- | --- | --- | --- | --- | --- | --- | --- | --- |
|  | **Naïve** | | **Vac** | | **Naïve** | | **Vac** | | |
| ***cd4*** | 1.11 ± 0.45 | | 1.06 ± 0.27 | | 1.03 ± 0.37 | | 1.20 ± 0.61 | | |
| ***cd8a*** | 1.40 ± 1.14 | | 0.72 ± 0.33 | | 1.28 ± 0.86 | | 0.91 ± 0.45 | | |
| ***cd8b*** | 1.18 ± 0.72 | | 0.55 ± 0.22 | | 1.26 ± 0.80 | | 0.77 ± 0.60 | | |
| ***igm*** | 1.04 ± 0.34 | | 0.8 ± 0.37 | | 0.88 ± 0.43 | | 0.78 ± 0.16 | | |
| ***igt*** | 4.63 ± 4.40 | | 3.29 ± 2.30 | | 5.60 ± 5.38 | | 5.59 ± 4.91 | | |
| ***il1b*** | 1.39 ± 1.19 | | 1.65 ± 2.09 | | 1.19 ± 0.59 | | 1.20 ± 0.82 | | |
| ***il8*** | 1.00 ± 0.15 | | 1.23 ± 0.41 | | 0.91 ± 0.22 | | 1.13 ± 0.47 | | |
| ***il10*** | 2.25 ± 3.94 | | 1.02 ± 0.66 | | 1.74 ± 1.05 | | 3.11 ± 4.50 | | |
| ***mhci*** | 1.65 ± 1.25 | | 1.65 ± 1.63 | | 1.74 ± 2.07 | | 2.03 ± 2.10 | | |
| ***migd*** | 1.03 ± 0.28 | | 1.23 ± 0.32 | | 1.08 ± 0.30 | | 1.15 ± 0.42 | | |
| ***tlr1*** | 1.00 ± 0.16 | | 1.02 ± 0.15 | | 0.92 ± 0.14 | | 0.91 ± 0.17 | | |
| ***saa*** | 3.41 ± 5.87 | | 3.97 ± 9.52 | | 1.43 ± 1.31 | | 2.68 ± 2.96 | | |
| ***tnfa*** | 1.02 ± 0.26 | | 1.17 ± 0.37 | | 1.03 ± 0.25 | | 1.21 ± 0.11 | | |
|  | |  | |  | |  | | **Vaccination** | |
|  | | **Diet** | | **Vaccination** | | **Diet × vaccination** | | **Naïve** | **Vac** |
| ***cd4*** | | ns | | ns | | ns | | - | - |
| ***cd8a*** | | ns | | ns | | ns | | - | - |
| ***cd8b*** | | ns | | 0.017 | | ns | | x | y |
| ***igm*** | | ns | | ns | | ns | | - | - |
| ***igt*** | | ns | | ns | | ns | | - | - |
| ***il1b*** | | ns | | ns | | ns | | - | - |
| ***il8*** | | ns | | ns | | ns | | - | - |
| ***il10*** | | ns | | ns | | ns | | - | - |
| ***mhci*** | | ns | | ns | | ns | | - | - |
| ***migd*** | | ns | | ns | | ns | | - | - |
| ***tlr1*** | | ns | | ns | | ns | | - | - |
| ***saa*** | | ns | | ns | | ns | | - | - |
| ***tnfa*** | | ns | | ns | | ns | | - | - |

**Table S7**. Effects of diet, time, vaccination and diet × vaccination interaction on the proportion of Yersinia ruckeri-positive fish. “x” and “y” stand for differences between naïve and vaccinated fish, and capital letters denote differences between experimental groups. *P*-values from Chi-square test and Fisher’s exact test; *p* ≤ 0.05.

|  |  |  |  | **Vaccination** | | **Diet × Vaccination** | | | |
| --- | --- | --- | --- | --- | --- | --- | --- | --- | --- |
|  | **χ²** | **df** | **p-value** | **Naïve** | **Vac** | **CTRL naïve** | **MET naïve** | **CTRL vac** | **MET vac** |
| **Diet** | 0.267 | 1 | ns | - | - | - | - | - | - |
| **Time** | 5.200 | 2 | ns | - | - | - | - | - | - |
| **Vaccination** | 9.600 | 1 | 0.002 | x | y | - | - | - | - |
| **Diet × Vaccination** | 10.933 | 3 | 0.012 | - | - | AB | A | B | B |

**Table S8.** Differences in the proportion of *Yersinia ruckeri*-positive fish between experimental groups within specific time points. P-values from Fisher’s exact test; p ≤ 0.05.

| **Time** | **CTRL naïve vs MET naïve** | **CTRL vac vs MET vac** | **CTRL naïve vs CTRL vac** | **MET naïve vs MET vac** | **CTRL naïve vs MET vac** | **MET naïve vs CTRL vac** |
| --- | --- | --- | --- | --- | --- | --- |
| **4 hpi** | ns | ns | ns | ns | ns | ns |
| **24 hpi** | ns | ns | ns | ns | ns | ns |
| **48 hpi** | ns | ns | ns | ns | ns | ns |


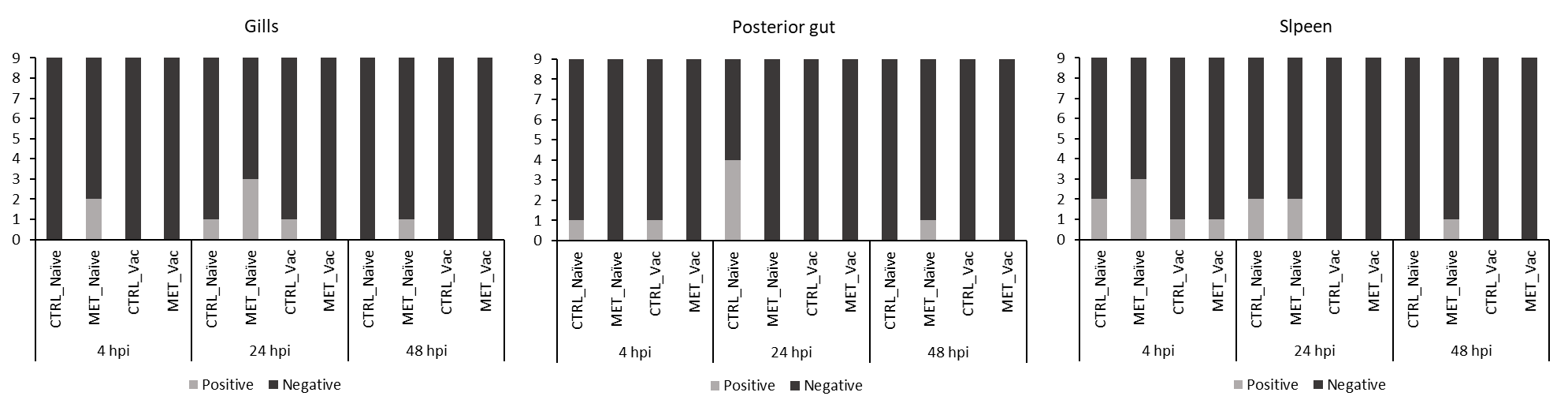


**Figure S1**. Number of gills, posterior gut and spleen samples with detectable Y. ruckeri at 4, 24 and 48 hours post-infection. Fish were fed CTRL or MET diets for 7 weeks (4 weeks before vaccination and 3 weeks post-vaccination) prior to infection with Y. ruckeri (5 × 10^5^ CFU/mL). Samples were considered positive when bacterial load was ≥ 230 CFU/mL.

**Table S9.** Effects of diet, vaccination, time, tissue, and diet × vaccination interaction on bacterial load. Lower case letters indicate differences across time points and capital letters denote differences between experimental groups. *P*-values from Kruskal-Wallis test followed by Bonferroni-corrected pairwise comparisons, or Mann-Whitney U test; *p* ≤ 0.05.

|  |  | **Time** | | | **Diet × Vaccination** | | | |
| --- | --- | --- | --- | --- | --- | --- | --- | --- |
|  | ***p*-value** | **4 hpi** | **24 hpi** | **48 hpi** | **CTRL naïve** | **MET naïve** | **CTRL vac** | **MET vac** |
| **Diet** | ns | - | - | - | - | - | - | - |
| **Time** | 0.014 | ab | a | b | - | - | - | - |
| **Tissue** | ns | - | - | - | - | - | - | - |
| **Vaccination** | < 0.001 | - | - | - | - | - | - | - |
| **Diet × Vaccination** | 0.001 | - | - | - | AB | A | B | B |

**Table S10.** Differences in bacterial load between experimental groups within specific tissues and time points. P-values from Mann-Whitney U test; p ≤ 0.05.

| **Tissue** | **Time** | **CTRL naïve vs MET naïve** | **CTRL vac vs MET vac** | **CTRL naïve vs CTRL vac** | **MET naïve vs MET vac** | **CTRL naïve vs MET vac** | **MET naïve vs CTRL vac** |
| --- | --- | --- | --- | --- | --- | --- | --- |
| **Gills** | **4 hpi** | ns | ns | ns | ns | ns | ns |
|  | **24 hpi** | ns | ns | ns | ns | ns | ns |
|  | **48 hpi** | ns | ns | ns | ns | ns | ns |
| **Gut** | **4 hpi** | ns | ns | ns | ns | ns | ns |
|  | **24 hpi** | 0.019 | ns | 0.019 | ns | 0.019 | ns |
|  | **48 hpi** | ns | ns | ns | ns | ns | ns |
| **Spleen** | **4 hpi** | ns | ns | ns | ns | ns | ns |
|  | **24 hpi** | ns | ns | ns | ns | ns | ns |
|  | **48 hpi** | ns | ns | ns | ns | ns | ns |

**Table S11.** Hematocrit, hemoglobin, mean corpuscular volume (MCV), mean corpuscular hemoglobin (MCH), mean corpuscular hemoglobin concentration (MCHC), red blood cells (RBC) and white blood cells (WBC) in rainbow trout fed dietary treatments for 7 weeks (4 weeks before vaccination and 21 days post-vaccination) and at 4, 24 and 48 hpi. Values are presented as mean ± SD (n = 8 at 21d and n = 9 at 4, 24 and 48 hpi). Different capital letters stand for differences between dietary treatments, different lowercase letters indicate differences among time points, and “x” and “y” denote differences between naïve (Nv) and vaccinated fish (Vac) (three-way ANOVA; *p* ≤ 0.05).

| **Parameters** | | |  | | | **Time** | | **CTRL** | | | | | | | **MET** | | | | | | | | | | | | |
| --- | --- | --- | --- | --- | --- | --- | --- | --- | --- | --- | --- | --- | --- | --- | --- | --- | --- | --- | --- | --- | --- | --- | --- | --- | --- | --- | --- |
|  | | |  | | |  | | **Naïve** | | | **Vac** | | | | **Naïve** | | | | | | **Vac** | | | | | | |
| **Hematocrit** | | | **(%)** | | | **21d** | | 44.75 ± 3.69 | | | 45.13 ± 3.56 | | | | 42.88 ± 3.64 | | | | | | 46.13 ± 4.02 | | | | | | |
|  |  |  |  |  |  | **4 hpi** | | 44.22 ± 5.38 | | | 46.89 ± 3.66 | | | | 46.67 ± 4.85 | | | | | | 47.11 ± 4.01 | | | | | | |
|  |  |  |  |  |  | **24 hpi** | | 46.22 ± 6.80 | | | 46.13 ± 1.25 | | | | 46.11 ± 6.68 | | | | | | 45.13 ± 1.64 | | | | | | |
|  |  |  |  |  |  | **48 hpi** | | 43.25 ± 1.04 | | | 50.67 ± 3.87 | | | | 45.44 ± 4.95 | | | | | | 51.33 ± 7.78 | | | | | | |
| **Hemoglobin** | | | **(g/dL)** | | | **21d** | | 1.28 ± 0.36 | | | 1.46 ± 0.20^ab^ | | | | 1.52 ± 0.08 | | | | | | 1.44 ± 0.23^ab^ | | | | | | |
|  |  |  |  |  |  | **4 hpi** | | 1.31 ± 0.20^y^ | | | 1.55 ± 0.25^ab,A,x^ | | | | 1.50 ± 0.32 | | | | | | 1.36 ± 0.11^b,B^ | | | | | | |
|  |  |  |  |  |  | **24 hpi** | | 1.54 ± 0.59 | | | 1.40 ± 0.15^b,B^ | | | | 1.50 ± 0.30 | | | | | | 1.71 ± 0.37^ab,A^ | | | | | | |
|  |  |  |  |  |  | **48 hpi** | | 1.33 ± 0.16 | | | 1.77 ± 0.31^a^ | | | | 1.65 ± 0.23 | | | | | | 1.75 ± 0.27^a^ | | | | | | |
| **MCV** | | | **(µm^3^)** | | | **21d** | | 596.28 ± 143.54 | | | 662.02 ± 140.96 | | | | 534.35 ± 67.67 | | | | | | 709.63 ± 203.54 | | | | | | |
|  |  |  |  |  |  | **4 hpi** | | 704.56 ± 175.72 | | | 538.03 ± 60.42 | | | | 615.39 ± 140.42 | | | | | | 669.58 ± 97.86 | | | | | | |
|  |  |  |  |  |  | **24 hpi** | | 665.24 ± 77.44 | | | 626.18 ± 70.72 | | | | 714.66 ± 124.03 | | | | | | 661.51 ± 124.84 | | | | | | |
|  |  |  |  |  |  | **48 hpi** | | 629.80 ± 117.11 | | | 643.73 ± 95.50 | | | | 642.31 ± 124.39 | | | | | | 634.81 ± 110.83 | | | | | | |
| **MCH** | | | **(pg/cell)** | | | **21d** | | 18.73 ± 4.20 | | | 21.64 ± 5.86 | | | | 19.78 ± 5.87 | | | | | | 24.10 ± 7.44 | | | | | | |
|  |  |  |  |  |  | **4 hpi** | | 20.56 ± 3.85 | | | 17.76 ± 2.78 | | | | 19.50 ± 4.71 | | | | | | 19.27 ± 2.43 | | | | | | |
|  |  |  |  |  |  | **24 hpi** | | 23.94 ± 3.44 | | | 19.41 ± 1.42 | | | | 22.99 ± 3.30 | | | | | | 24.18 ± 4.83 | | | | | | |
|  |  |  |  |  |  | **48 hpi** | | 20.54 ± 4.24 | | | 22.44 ± 4.97 | | | | 23.34 ± 4.92 | | | | | | 22.45 ± 4.09 | | | | | | |
| **MCHC** | | | **(g/100 mL)** | | | **21d** | | 2.86 ± 0.73 | | | 3.25 ± 0.40 | | | | 3.37 ± 0.50 | | | | | | 3.11 ± 0.44ab | | | | | | |
|  |  |  |  |  |  | **4 hpi** | | 2.99 ± 0.48^B^ | | | 3.31 ± 0.41^A^ | | | | 3.38 ± 0.24^A,x^ | | | | | | 2.89 ± 0.26^b,B,y^ | | | | | | |
|  |  |  |  |  |  | **24 hpi** | | 3.62 ± 0.49^x^ | | | 3.00 ± 0.31^B,y^ | | | | 3.24 ± 0.26 | | | | | | 3.72 ± 0.83^a,A^ | | | | | | |
|  |  |  |  |  |  | **48 hpi** | | 3.40 ± 0.98 | | | 3.49 ± 0.55 | | | | 3.66 ± 0.53 | | | | | | 3.48 ± 0.69^ab^ | | | | | | |
| **RBC** | | | **(× 10^6^/µL)** | | | **21d** | | 0.80 ± 0.25 | | | 0.70 ± 0.15 | | | | 0.76 ± 0.19 | | | | | | 0.68 ± 0.16 | | | | | | |
|  |  |  |  |  |  | **4 hpi** | | 0.65 ± 0.12 | | | 0.87 ± 0.10 | | | | 0.79 ± 0.19 | | | | | | 0.71 ± 0.08 | | | | | | |
|  |  |  |  |  |  | **24 hpi** | | 0.62 ± 0.06 | | | 0.72 ± 0.11 | | | | 0.66 ± 0.18 | | | | | | 0.71 ± 0.13 | | | | | | |
|  |  |  |  |  |  | **48 hpi** | | 0.72 ± 0.11 | | | 0.80 ± 0.12 | | | | 0.72 ± 0.13 | | | | | | 0.78 ± 0.07 | | | | | | |
| **WBC** | | | **(× 10^4^/µL)** | | | **21d** | | 4.79 ± 1.46 | | | 4.59 ± 0.86 | | | | 4.44 ± 0.78 | | | | | | 4.19 ± 0.74 | | | | | | |
|  |  |  |  |  |  | **4 hpi** | | 4.18 ± 1.37 | | | 3.76 ± 1.30 | | | | 3.06 ± 1.31 | | | | | | 3.84 ± 0.91 | | | | | | |
|  |  |  |  |  |  | **24 hpi** | | 3.08 ± 0.87 | | | 4.03 ± 0.90 | | | | 2.84 ± 1.25 | | | | | | 4.19 ± 0.87 | | | | | | |
|  |  |  |  |  |  | **48 hpi** | | 3.21 ± 0.67 | | | 4.34 ± 1.10 | | | | 3.40 ± 1.29 | | | | | | 3.29 ± 0.63 | | | | | | |
|  |  |  | |  |  | |  | |  |  | |  | | | | |  | | **Time × Vaccination** | | | | | | | | |
|  |  |  | |  |  | |  | |  |  | | **Time** | | | | | **Vac** | | **21d** | | | **4 hpi** | | **24 hpi** | | **48 hpi** | |
|  | **Time (T)** | **Diet (D)** | | **Vaccination (V)** | **T × D** | | **T × V** | | **D × V** | **T × D × V** | | **21d** | **4 hpi** | **24 hpi** | | **48 hpi** | **Nv** | **Vac** | **Nv** | **Vac** | | **Nv** | **Vac** | **Nv** | **Vac** | **Nv** | **Vac** |
| **Hematocrit** | ns | ns | | 0.003 | ns | | 0.035 | | ns | ns | | - | - | - | | - | - | - | - | b | | - | b | - | b | y | a,x |
| **Hemoglobin** | 0.015 | ns | | 0.042 | ns | | ns | | ns | 0.027 | | - | - | - | | - | - | - | - | - | | - | - | - | - | - | - |
| **MCV** | ns | ns | | ns | ns | | 0.016 | | ns | ns | | - | - | - | | - | - | - | b,y | x | | ab | - | a | - | ab | - |
| **MCH** | 0.004 | ns | | ns | ns | | ns | | ns | ns | | ab | b | a | | a | - | - | - | - | | - | - | - | - | - | - |
| **MCHC** | 0.014 | ns | | ns | ns | | ns | | ns | 0.001 | | - | - | - | | - | - | - | - | - | | - | - | - | - | - | - |
| **RBC** | ns | ns | | ns | ns | | ns | | ns | ns | | - | - | - | | - | - | - | - | - | | - | - | - | - | - | - |
| **WBC** | < 0.001 | ns | | 0.025 | ns | | ns | | ns | ns | | a | b | b | | b | y | x | - | - | | - | - | - | - | - | - |

**Table S12.** Absolute values of peripheral blood leucocytes (thrombocytes, lymphocytes, monocytes and neutrophils) in rainbow trout fed dietary treatments for 7 weeks (4 weeks before vaccination and 21 days post-vaccination) and at 4, 24 and 48 hpi. Values are presented as mean ± SD (n = 8 at 21d and n = 9 at 4, 24 and 48 hpi). Different lowercase letters indicate differences among time points, and “x” and “y” denote differences between naïve and vaccinated fish (three-way ANOVA; *p* ≤ 0.05).

| **Parameters** | | | |  | | **Time** | | **CTRL** | | | | **MET** | | | | | | | |
| --- | --- | --- | --- | --- | --- | --- | --- | --- | --- | --- | --- | --- | --- | --- | --- | --- | --- | --- | --- |
|  | | | |  | |  | | **Naïve** | | **Vac** | | **Naïve** | | | | **Vac** | | | |
| **Thrombocytes** | | | | **(× 10^4^/µL)** | | **21d** | | 0.74 ± 0.36 | | 0.69 ± 0.35 | | 0.82 ± 0.62 | | | | 0.36 ± 0.06 | | | |
|  |  |  |  |  |  | **4 hpi** | | 1.19 ± 0.44 | | 1.15 ± 0.64 | | 0.66 ± 0.29 | | | | 0.74 ± 0.44 | | | |
|  |  |  |  |  |  | **24 hpi** | | 0.82 ± 0.31 | | 0.80 ± 0.62 | | 0.85 ± 0.51 | | | | 0.71 ± 0.27 | | | |
|  |  |  |  |  |  | **48 hpi** | | 1.03 ± 0.36 | | 1.38 ± 0.39 | | 1.21 ± 0.52 | | | | 1.29 ± 0.66 | | | |
| **Lymphocytes** | | | |  |  | **21d** | | 3.75 ± 1.59 | | 3.55 ± 0.59 | | 3.28 ± 0.70 | | | | 3.49 ± 0.80 | | | |
|  |  |  |  |  |  | **4 hpi** | | 2.67 ± 1.12 | | 2.39 ± 1.09 | | 2.05 ± 1.17 | | | | 2.61 ± 0.94 | | | |
|  |  |  |  |  |  | **24 hpi** | | 1.88 ± 0.59 | | 2.66 ± 0.83 | | 1.48 ± 0.73 | | | | 2.79 ± 1.03 | | | |
|  |  |  |  |  |  | **48 hpi** | | 1.75 ± 0.58 | | 2.60 ± 1.05 | | 1.86 ± 0.83 | | | | 1.71 ± 0.83 | | | |
| **Monocytes** | | | |  |  | **21d** | | 0.08 ± 0.06 | | 0.10 ± 0.05 | | 0.11 ± 0.13 | | | | 0.11 ± 0.06 | | | |
|  |  |  |  |  |  | **4 hpi** | | 0.13 ± 0.11 | | 0.05 ± 0.04 | | 0.10 ± 0.11 | | | | 0.09 ± 0.08 | | | |
|  |  |  |  |  |  | **24 hpi** | | 0.15 ± 0.09 | | 0.21 ± 0.07 | | 0.15 ± 0.11 | | | | 0.22 ± 0.12 | | | |
|  |  |  |  |  |  | **48 hpi** | | 0.11 ± 0.08 | | 0.15 ± 0.08 | | 0.09 ± 0.03 | | | | 0.07 ± 0.04 | | | |
| **Neutrophils** | | | |  |  | **21d** | | 0.21 ± 0.18 | | 0.30 ± 0.20 | | 0.22 ± 0.21 | | | | 0.24 ± 0.09 | | | |
|  |  |  |  |  |  | **4 hpi** | | 0.19 ± 0.14 | | 0.17 ± 0.05 | | 0.13 ± 0.06 | | | | 0.19 ± 0.22 | | | |
|  |  |  |  |  |  | **24 hpi** | | 0.29 ± 0.21 | | 0.31 ± 0.14 | | 0.21 ± 0.13 | | | | 0.42 ± 0.27 | | | |
|  |  |  |  |  |  | **48 hpi** | | 0.32 ± 0.17 | | 0.23 ± 0.15 | | 0.22 ± 0.11 | | | | 0.21 ± 0.17 | | | |
|  |  |  |  | |  | |  | |  | |  | | **Time** | | | | | **Vaccination** | |
|  | **Time (T)** | **Diet (D)** | **Vaccination (V)** | | **T × D** | | **T × V** | | **D × V** | | **T × D × V** | | **21d** | **4 hpi** | **24 hpi** | | **48 hpi** | **Naïve** | **Vac** |
| **Thrombocytes** | < 0.001 | ns | ns | | ns | | ns | | ns | | ns | | b | ab | b | | a | - | - |
| **Lymphocytes** | < 0.001 | ns | 0.020 | | ns | | ns | | ns | | ns | | a | b | b | | b | y | x |
| **Monocytes** | < 0.001 | ns | ns | | ns | | ns | | ns | | ns | | b | b | a | | b | - | - |
| **Neutrophils** | 0.009 | ns | ns | | ns | | ns | | ns | | ns | | ab | b | a | | ab | - | - |

**Table S13.** Triglycerides (TAG), cholesterol, glucose, glycogen and lactate levels in the liver of rainbow trout fed dietary treatments for 7 weeks (4 weeks before vaccination and 21 days post-vaccination) and at 4, 24 and 48 hpi. Values are presented as mean ± SD (n = 8 at 21d and n = 9 at 4, 24 and 48 hpi). Different lowercase letters indicate differences among time points, and “x” and “y” denote differences between naïve (Nv) and vaccinated fish (Vac) (three-way ANOVA; *p* ≤ 0.05).

| **Parameters** |  | **Time** | **CTRL** | | **MET** | |
| --- | --- | --- | --- | --- | --- | --- |
|  |  |  | **Naïve** | **Vac** | **Naïve** | **Vac** |
| **TAG** | **(mg/g ww )** | **21d** | 21.44 ± 1.60 | 18.92 ± 4.83 | 20.58 ± 1.55 | 21.92 ± 5.53 |
|  |  | **4 hpi** | 18.20 ± 3.13 | 19.31 ± 4.47 | 20.89 ± 4.78 | 18.84 ± 4.86 |
|  |  | **24 hpi** | 24.25 ± 5.24 | 24.14 ± 6.65 | 26.15 ± 5.58 | 22.76 ± 4.48 |
|  |  | **48 hpi** | 24.25 ± 4.27 | 25.90 ± 4.47 | 25.93 ± 3.38 | 24.59 ± 2.68 |
| **Cholesterol** |  | **21d** | 0.46 ± 0.13 | 0.44 ± 0.05 | 0.44 ± 0.04 | 0.46 ± 0.09 |
|  |  | **4 hpi** | 0.43 ± 0.08 | 0.53 ± 0.01 | 0.48 ± 0.08 | 0.49 ± 0.15 |
|  |  | **24 hpi** | 0.55 ± 0.11 | 0.52 ± 0.13 | 0.55 ± 0.09 | 0.52 ± 0.12 |
|  |  | **48 hpi** | 0.44 ± 0.11 | 0.56 ± 0.11 | 0.52 ± 0.07 | 0.49 ± 0.10 |
| **Glucose** |  | **21d** | 45.45 ± 14.84 | 38.31 ± 16.60 | 39.95 ± 16.19 | 35.77 ± 14.47 |
|  |  | **4 hpi** | 51.42 ± 5.93 | 62.64 ± 17.75 | 67.16 ± 15.27 | 62.23 ± 20.41 |
|  |  | **24 hpi** | 53.55 ± 29.88 | 60.65 ± 16.35 | 54.21 ± 9.09 | 64.18 ± 35.76 |
|  |  | **48 hpi** | 51.81 ± 17.09 | 61.49 ± 11.68 | 57.39 ± 13.16 | 64.46 ± 17.20 |
| **Glycogen** |  | **21d** | 144.19 ± 79.22 | 231.82 ± 82.75 | 167.98 ± 108.89 | 195.00 ± 113.33 |
|  |  | **4 hpi** | 250.88 ± 114.25 | 293.10 ± 86.43 | 307.51 ± 121.20 | 247.72 ± 70.24 |
|  |  | **24 hpi** | 209.41 ± 100.13 | 222.80 ± 98.41 | 201.79 ± 78.38 | 182.07 ± 69.96 |
|  |  | **48 hpi** | 129.44 ± 57.78 | 142.83 ± 56.15 | 149.25 ± 98.03 | 147.85 ± 95.03 |
| **Lactate** |  | **21d** | 0.25 ± 0.05 | 0.20 ± 0.09 | 0.25 ± 0.04 | 0.23 ± 0.07 |
|  |  | **4 hpi** | 0.25 ± 0.06 | 0.25 ± 0.05 | 0.22 ± 0.05 | 0.23 ± 0.06 |
|  |  | **24 hpi** | 0.24 ± 0.06 | 0.22 ± 0.07 | 0.24 ± 0.06 | 0.23 ± 0.06 |
|  |  | **48 hpi** | 0.19 ± 0.05 | 0.28 ± 0.07 | 0.21 ± 0.06 | 0.27 ± 0.08 |

|  |  |  |  |  |  |  |  |  | | | | **Time × Vaccination** | | | | | | | |
| --- | --- | --- | --- | --- | --- | --- | --- | --- | --- | --- | --- | --- | --- | --- | --- | --- | --- | --- | --- |
|  |  |  |  |  |  |  |  | **Time** | | | | **21d** | | **4 hpi** | | **24 hpi** | | **48 hpi** | |
|  | **T (T)** | **Diet (D)** | **Vaccination (V)** | **T× D** | **T × V** | **D × V** | **T × D × V** | **21d** | **4 hpi** | **24 hpi** | **48 hpi** | **Nv** | **Vac** | **Nv** | **Vac** | **Nv** | **Vac** | **Nv** | **Vac** |
| **TAG** | < 0.001 | ns | ns | ns | ns | ns | ns | b | b | a | a | - | - | - | - | - | - | - | - |
| **Cholesterol** | 0.012 | ns | ns | ns | ns | ns | ns | b | ab | a | ab | - | - | - | - | - | - | - | - |
| **Glucose** | < 0.001 | ns | ns | ns | ns | ns | ns | b | a | a | a | - | - | - | - | - | - | - | - |
| **Glycogen** | < 0.001 | ns | ns | ns | ns | ns | ns | bc | a | b | c | - | - | - | - | - | - | - | - |
| **Lactate** | ns | ns | ns | ns | 0.003 | ns | ns | - | - | - | - | a | - | ab | - | ab | - | b,y | x |

**Table S14.** Catalase activity (CAT), superoxide dismutase (SOD), total glutathione (tGSH), reduced glutathione (rGSH), oxidized glutathione (GSSG) and lipid peroxidation levels (LPO) in the liver of rainbow trout fed dietary treatments for 7 weeks (4 weeks before vaccination and 21 days post-vaccination) and at 4, 24 and 48 hpi. Values are presented as mean ± SD (n = 8 at 21d and n = 9 at 4, 24 and 48 hpi). Different capital letters stand for differences between dietary treatments, different lowercase letters indicate differences among time points, and “x” and “y” denote differences between naïve (Nv) and vaccinated fish (Vac) (three-way ANOVA; *p* ≤ 0.05).

| **Parameters** | | |  | | | | **Time** | | | **CTRL** | | | | | | | | | | | | **MET** | | | | | | | | | | | | | |
| --- | --- | --- | --- | --- | --- | --- | --- | --- | --- | --- | --- | --- | --- | --- | --- | --- | --- | --- | --- | --- | --- | --- | --- | --- | --- | --- | --- | --- | --- | --- | --- | --- | --- | --- | --- |
|  | | |  | | | |  | | | **Naïve** | | | | | | | **Vac** | | | | | **Naïve** | | | | | | **Vac** | | | | | | | |
| **CAT** | | | **(units/mg protein)** | | | | **21d** | | | 161.52 ± 43.76 | | | | | | | 211.01 ± 39.23 | | | | | 156.93 ± 30.72 | | | | | | 161.55 ± 60.02 | | | | | | | |
|  |  |  |  |  |  |  | **4 hpi** | | | 213.92 ± 52.30 | | | | | | | 151.38 ± 49.83 | | | | | 219.93 ± 58.74 | | | | | | 123.19 ± 24.02 | | | | | | | |
|  |  |  |  |  |  |  | **24 hpi** | | | 185.22 ± 39.97 | | | | | | | 198.95 ± 39.91 | | | | | 182.07 ± 33.94 | | | | | | 190.33 ± 53.46 | | | | | | | |
|  |  |  |  |  |  |  | **48 hpi** | | | 201.79 ± 63.76 | | | | | | | 214.06 ± 48.09 | | | | | 133.79 ± 37.70 | | | | | | 229.21 ± 92.75 | | | | | | | |
| **SOD** | | | **(units/mg protein)** | | | | **21d** | | | 95.58 ± 9.78 | | | | | | | 78.79 ± 15.76 | | | | | 92.78 ± 17.99 | | | | | | 86.98 ± 9.55 | | | | | | | |
|  |  |  |  |  |  |  | **4 hpi** | | | 88.32 ± 11.14 | | | | | | | 95.21 ± 19.84 | | | | | 77.87 ± 10.38 | | | | | | 90.30 ± 6.66 | | | | | | | |
|  |  |  |  |  |  |  | **24 hpi** | | | 72.23 ± 20.41 | | | | | | | 88.14 ± 13.70 | | | | | 67.78 ± 18.53 | | | | | | 77.14 ± 8.57 | | | | | | | |
|  |  |  |  |  |  |  | **48 hpi** | | | 46.67 ± 7.74 | | | | | | | 65.89 ± 10.07 | | | | | 37.80 ± 8.15 | | | | | | 52.62 ± 15.49 | | | | | | | |
| **tGSH** | | | **(µM)** | | | | **21d** | | | 1246.72 ± 363.38 | | | | | | | 1324.06 ± 183.06 | | | | | 1377.75 ± 220.90 | | | | | | 1368.05 ± 313.32 | | | | | | | |
|  |  |  |  |  |  |  | **4 hpi** | | | 1384.56 ± 189.63 | | | | | | | 1222.31 ± 236.82 | | | | | 1490.48 ± 282.94 | | | | | | 1330.34 ± 226.84 | | | | | | | |
|  |  |  |  |  |  |  | **24 hpi** | | | 1263.59 ± 128.77 | | | | | | | 1269.31 ± 127.56 | | | | | 1239.08 ± 242.73 | | | | | | 1358.99 ± 95.41 | | | | | | | |
|  |  |  |  |  |  |  | **48 hpi** | | | 1260.44 ± 242.48 | | | | | | | 1219.73 ± 160.31 | | | | | 1117.52 ± 269.66 | | | | | | 1012.77 ± 198.82 | | | | | | | |
| **rGSH** | | | **(µM)** | | | | **21d** | | | 849.44 ± 317.94 | | | | | | | 977.39 ± 250.56 | | | | | 980.69 ± 201.19 | | | | | | 992.78 ± 220.70 | | | | | | | |
|  |  |  |  |  |  |  | **4 hpi** | | | 1008.30 ± 171.10 | | | | | | | 856.28 ± 173.99 | | | | | 1176.32 ± 259.97 | | | | | | 966.15 ± 94.46 | | | | | | | |
|  |  |  |  |  |  |  | **24 hpi** | | | 1002.12 ± 134.33 | | | | | | | 959.12 ± 129.33 | | | | | 934.85 ± 259.16 | | | | | | 1062.73 ± 97.34 | | | | | | | |
|  |  |  |  |  |  |  | **48 hpi** | | | 971.79 ± 214.78 | | | | | | | 927.71 ± 149.88 | | | | | 924.22 ± 237.01 | | | | | | 774.28 ± 107.15 | | | | | | | |
| **GSSG** | | | **(µM)** | | | | **21d** | | | 198.64 ± 51.24 | | | | | | | 173.33 ± 79.73 | | | | | 198.73 ± 33.07 | | | | | | 187.64 ± 59.02 | | | | | | | |
|  |  |  |  |  |  |  | **4 hpi** | | | 188.13 ± 38.81 | | | | | | | 183.02 ± 40.04 | | | | | 157.08 ± 33.42 | | | | | | 155.41 ± 56.90 | | | | | | | |
|  |  |  |  |  |  |  | **24 hpi** | | | 130.77 ± 40.03 | | | | | | | 155.10 ± 33.21 | | | | | 152.11 ± 42.64 | | | | | | 132.28 ± 19.64 | | | | | | | |
|  |  |  |  |  |  |  | **48 hpi** | | | 136.85 ± 37.72 | | | | | | | 146.01 ± 27.45 | | | | | 96.65 ± 28.03 | | | | | | 140.51 ± 35.85 | | | | | | | |
| **GSH/GSSG** | | |  | | | | **21d** | | | 4.41 ± 1.96 | | | | | | | 4.84 ± 1.36^ab^ | | | | | 5.06 ± 1.40^c^ | | | | | | 5.63 ± 1.48 | | | | | | | |
|  |  |  |  |  |  |  | **4 hpi** | | | 5.54 ± 1.32 | | | | | | | 4.77 ± 0.80^b^ | | | | | 7.74 ± 2.34^ab^ | | | | | | 7.55 ± 3.49 | | | | | | | |
|  |  |  |  |  |  |  | **24 hpi** | | | 8.30 ± 2.61 | | | | | | | 6.48 ± 1.91^ab^ | | | | | 7.12 ± 1.22^bc^ | | | | | | 7.31 ± 1.32 | | | | | | | |
|  |  |  |  |  |  |  | **48 hpi** | | | 7.24 ± 2.65 | | | | | | | 6.61 ± 0.52^a^ | | | | | 9.87 ± 2.42^a,x^ | | | | | | 5.41 ± 1.58^y^ | | | | | | | |
| **LPO** | | | **(nmol/g wt)** | | | | **21d** | | | 43.46 ± 5.80 | | | | | | | 42.34 ± 1.72 | | | | | 41.60 ± 4.48 | | | | | | 42.47 ± 4.34 | | | | | | | |
|  |  |  |  |  |  |  | **4 hpi** | | | 41.26 ± 3.64 | | | | | | | 44.82 ± 3.86 | | | | | 39.97 ± 3.58 | | | | | | 45.01 ± 2.16 | | | | | | | |
|  |  |  |  |  |  |  | **24 hpi** | | | 41.99 ± 3.00 | | | | | | | 42.03 ± 2.83 | | | | | 42.40 ± 2.87 | | | | | | 42.78 ± 3.48 | | | | | | | |
|  |  |  |  |  |  |  | **48 hpi** | | | 43.14 ± 5.42 | | | | | | | 43.14 ± 2.48 | | | | | 41.54 ± 2.35 | | | | | | 41.09 ± 3.83 | | | | | | | |
|  |  |  | |  |  |  | |  |  | |  | | | |  | | | **Time x Diet** | | | | | | | | | **Time × Vaccination** | | | | | | | | |
|  |  |  | |  |  |  | |  |  | | **Time** | | | | **Diet** | | | **21d** | | **4 hpi** | | | **24 hpi** | | **48 hpi** | | **21d** | | | **4 hpi** | | **24 hpi** | | **48 hpi** | |
|  | **Time**  **(T)** | **Diet**  **(D)** | | **Vaccination**  **(V)** | **T × D** | **T × V** | | **D × V** | **T × D × V** | | **21d** | **4 hpi** | **24 hpi** | **48 hpi** | **CTRL** | **MET** | | **CTRL** | **MET** | **CTRL** | **MET** | | **CTRL** | **MET** | **CTRL** | **MET** | **Nv** | | **Vac** | **Nv** | **Vac** | **Nv** | **Vac** | **Nv** | **Vac** |
| **CAT** | < 0.001 | ns | | ns | ns | < 0.001 | | ns | ns | | - | - | - | - | - | - | | - | - | - | - | | - | - | - | - | b | | a | a,x | b,y | ab | a | a | a |
| **SOD** | < 0.001 | 0.013 | | 0.008 | ns | 0.002 | | ns | ns | | - | - | - | - | A | B | | - | - | - | - | | - | - | - | - | a | | a | ab,y | a,x | b | a | c,y | b,x |
| **tGSH** | < 0.001 | ns | | ns | 0.045 | ns | | ns | ns | | - | - | - | - | - | - | | - | a | - | a | | - | a | A | b,B | - | | - | - | - | - | - | - | - |
| **rGSH** | ns | ns | | ns | ns | 0.030 | | ns | ns | | - | - | - | - | - | - | | - | - | - | - | | - | - | - | - | - | | - | x | y | - | - | - | - |
| **GSSG** | < 0.001 | ns | | ns | ns | ns | | ns | ns | | a | ab | bc | c | - | - | | - | - | - | - | | - | - | - | - | - | | - | - | - | - | - | - | - |
| **GSH/GSSG** | < 0.001 | 0.005 | | 0.029 | ns | 0.032 | | ns | 0.049 | | - | - | - | - | - | - | | - | - | - | - | | - | - | - | - | - | | - | - | - | - | - | - | - |
| **LPO** | ns | ns | | ns | ns | 0.034 | | ns | ns | | - | - | - | - | - | - | | - | - | - | - | | - | - | - | - | - | | - | y | x | - | - | - | - |

**Table S15.** Quantitative expression of *bhmt*, *cd8b*, *gdh*, *hoad*, *leap2*, *migd*, *sahh1*, *amd1*, *sat1*, *sms*, *tlr1* and *tlr5* in the liver of rainbow trout fed dietary treatments for 7 weeks (4 weeks before vaccination and 21 days post-vaccination) and at 4, 24 and 48 hpi. Values are presented as mean ± SD (n = 8 at 21d and n = 9 at 4, 24 and 48 hpi). Different capital letters stand for differences between dietary treatments, different lowercase letters indicate differences among time points, and “x” and “y” denote differences between naïve and vaccinated fish (three-way ANOVA; *p* ≤ 0.05).

| **Parameters** | **Time** | **CTRL** | | **MET** | |
| --- | --- | --- | --- | --- | --- |
|  |  | **Naïve** | **Vac** | **Naïve** | **Vac** |
| ***bhmt*** | **21 d** | 1.31 ± 1.11 | 1.67 ± 2.39 | 1.28 ± 0.62 | 0.67 ± 0.36 |
|  | **4 hpi** | 0.89 ± 0.56 | 2.07 ± 2.93 | 1.36 ± 1.11 | 0.51 ± 0.81 |
|  | **24 hpi** | 1.42 ± 1.14 | 1.31 ± 0.33 | 1.17 ± 0.77 | 0.65 ± 0.43 |
|  | **48 hpi** | 7.84 ± 7.18 | 7.34 ± 6.03 | 3.54 ± 2.35 | 4.13 ± 2.49 |
| ***cd8b*** | **21 d** | 1.16 ± 0.76 | 1.20 ± 1.15 | 1.13 ± 0.87 | 1.14 ± 1.10 |
|  | **4 hpi** | 1.18 ± 0.88 | 1.13 ± 0.90 | 1.14 ± 0.67 | 1.48 ± 0.44 |
|  | **24 hpi** | 0.50 ± 0.16 | 0.80 ± 0.45 | 0.80 ± 0.47 | 0.97 ± 0.72 |
|  | **48 hpi** | 0.57 ± 0.27 | 0.74 ± 0.50 | 1.08±1.01 | 1.01 ± 0.59 |
| ***gdh*** | **21 d** | 1.17 ± 0.63^c^ | 0.83 ± 0.49^c^ | 0.78 ± 0.40^b^ | 1.19 ± 0.59^b^ |
|  | **4 hpi** | 0.85 ± 0.39^c^ | 1.41 ± 0.73^c^ | 1.19 ± 0.64^ab^ | 1.85 ± 0.93^b^ |
|  | **24 hpi** | 2.41 ± 2.07^b^ | 2.99 ± 1.72^b^ | 3.33 ± 2.35^a,x^ | 1.47 ± 0.82^b,y^ |
|  | **48 hpi** | 4.19 ± 1.64^a^ | 4.18 ± 2.58^a^ | 2.81 ± 2.34^a^ | 4.98 ± 3.47^a^ |
| ***hoad*** | **21 d** | 1.02 ± 0.26 | 1.11 ± 0.38 | 1.07 ± 0.27 | 1.24 ± 0.39 |
|  | **4 hpi** | 1.15 ± 0.28 | 1.41 ± 0.24 | 1.12 ± 0.27 | 1.32 ± 0.28 |
|  | **24 hpi** | 1.00 ± 0.18 | 1.25 ± 0.58 | 1.12 ± 0.50 | 1.04 ± 0.40 |
|  | **48 hpi** | 2.07 ± 0.40 | 2.04 ± 0.86 | 1.76 ± 1.07 | 2.19 ± 0.96 |
| ***leap2*** | **21 d** | 1.12 ± 0.61 | 1.29 ± 0.4 | 1.08 ± 0.31 | 1.35 ± 0.58 |
|  | **4 hpi** | 0.65 ± 0.29 | 0.80 ± 0.57 | 0.68 ± 0.19 | 0.48 ± 0.23 |
|  | **24 hpi** | 1.01 ± 0.95 | 1.35 ± 0.62 | 1.09 ± 0.76 | 0.77 ± 0.24 |
|  | **48 hpi** | 1.69 ± 0.57 | 2.34 ± 0.75 | 1.62 ± 1.38 | 1.49 ± 0.39 |
| ***migd*** | **21 d** | 1.10 ± 0.51 | 1.19 ± 0.61 | 0.76 ± 0.21 | 1.15 ± 0.53 |
|  | **4 hpi** | 1.19 ± 0.64 | 0.83 ± 0.36 | 0.85 ± 0.35 | 0.87 ± 0.34 |
|  | **24 hpi** | 0.62 ± 0.21 | 0.83 ± 0.43 | 0.69 ± 0.25 | 1.05 ± 0.50 |
|  | **48 hpi** | 0.87 ± 0.47 | 0.85 ± 0.52 | 0.63 ± 0.30 | 0.86 ± 0.62 |
| ***sahh*** | **21 d** | 1.24 ± 0.81 | 1.49 ± 1.35 | 1.22 ± 1.15 | 1.25 ± 1.13 |
|  | **4 hpi** | 0.91 ± 0.51 | 0.28 ± 0.11 | 1.18 ± 0.84 | 1.27 ± 0.99 |
|  | **24 hpi** | 0.30 ± 0.19 | 0.51 ± 0.32 | 0.53 ± 0.24 | 1.03 ± 0.75 |
|  | **48 hpi** | 0.58 ± 0.35 | 0.87 ± 0.69 | 1.01 ± 0.93 | 0.72 ± 0.68 |
| ***amd1*** | **21 d** | 1.32 ± 0.89 | 1.70 ± 0.73 | 1.00 ± 0.39 | 0.99 ± 0.49 |
|  | **4 hpi** | 1.66 ± 0.69 | 2.72 ± 1.08 | 1.77 ± 1.07 | 2.29 ± 1.52 |
|  | **24 hpi** | 3.00 ± 1.30 | 4.42 ± 2.08 | 2.94 ± 2.07 | 2.63 ± 1.59 |
|  | **48 hpi** | 2.36 ± 0.89 | 2.65 ± 0.64 | 2.47 ± 1.26 | 2.83 ± 1.52 |
| ***sat1*** | **21 d** | 1.11 ± 0.67 | 1.03 ± 0.45 | 0.98 ± 0.59 | 1.42 ± 0.41 |
|  | **4 hpi** | 1.76 ± 0.88 | 1.63 ± 0.55 | 1.59 ± 0.54 | 1.58 ± 0.46 |
|  | **24 hpi** | 1.14 ± 0.34 | 1.15 ± 0.45 | 1.19 ± 0.48 | 1.49 ± 0.36 |
|  | **48 hpi** | 1.51 ± 0.53 | 1.69 ± 0.95 | 1.22 ± 0.76 | 1.37 ± 0.32 |
| ***sms*** | **21 d** | 1.10 ± 0.49 | 1.66 ± 0.71 | 1.15 ± 0.58 | 1.20 ± 0.49 |
|  | **4 hpi** | 1.46 ± 0.15 | 0.97 ± 0.21 | 0.96 ± 0.30 | 0.91 ± 0.31 |
|  | **24 hpi** | 0.75 ± 0.42 | 1.02 ± 0.54 | 0.86 ± 0.32 | 1.69 ± 1.67 |
|  | **48 hpi** | 1.01 ± 0.40 | 1.02 ± 0.42 | 0.98 ± 0.37 | 1.26 ± 0.46 |
| ***tlr1*** | **21 d** | 1.22 ± 0.77 | 1.14 ± 0.66 | 0.89 ± 0.49 | 0.94 ± 0.33 |
|  | **4 hpi** | 0.81 ± 0.28 | 0.62 ± 0.34 | 0.76 ± 0.29 | 0.97 ± 0.26 |
|  | **24 hpi** | 0.78 ± 0.34 | 0.64 ± 0.18 | 0.63 ± 0.27 | 0.89 ± 0.31 |
|  | **48 hpi** | 0.67 ± 0.22 | 0.67 ± 0.26 | 0.67 ± 0.16 | 0.63 ± 0.22 |
| ***tlr5*** | **21 d** | 1.36 ± 1.05 | 0.96 ± 1.58 | 0.86 ± 1.04 | 1.78 ± 2.13 |
|  | **4 hpi** | 2.86 ± 3.15 | 0.94 ± 0.44 | 2.57 ± 2.61 | 4.03 ± 4.83 |
|  | **24 hpi** | 54.93 ± 71.10 | 4.32 ± 2.21 | 36.94 ± 57.80 | 6.88 ± 5.23 |
|  | **48 hpi** | 1.44 ± 2.92 | 0.72 ± 0.58 | 0.74 ± 0.62 | 0.21 ± 0.02 |

|  |  |  |  |  |  |  |  | **Time** | | | | **Diet** | | **Vaccination** | |
| --- | --- | --- | --- | --- | --- | --- | --- | --- | --- | --- | --- | --- | --- | --- | --- |
|  | **Time** | **Diet** | **Vaccination** | **Time × Diet** | **Time × Vaccination** | **Diet × Vaccination** | **Time × Diet × Vaccination** | **21d** | **4 hpi** | **24 hpi** | **48 hpi** | **CTRL** | **MET** | **Naïve** | **Vac** |
| ***bhmt*** | < 0.001 | ns | ns | ns | ns | ns | ns | b | b | b | a | - | - | - | - |
| ***cd8b*** | ns | ns | ns | ns | ns | ns | ns | - | - | - | - | - | - | - | - |
| ***gdh*** | < 0.001 | ns | ns | ns | ns | ns | 0.013 | - | - | - | - | - | - | - | - |
| ***hoad*** | < 0.001 | ns | ns | ns | ns | ns | ns | b | b | b | a | - | - | - | - |
| ***leap2*** | < 0.001 | ns | ns | ns | ns | ns | ns | b | c | bc | a | - | - | - | - |
| ***migd*** | ns | ns | ns | ns | ns | ns | ns | - | - | - | - | - | - | - | - |
| ***sahh*** | 0.009 | ns | ns | ns | ns | ns | ns | a | ab | b | ab | - | - | - | - |
| ***amd1*** | < 0.001 | 0.036 | 0.044 | ns | ns | ns | ns | c | b | a | ab | A | B | y | x |
| ***sat1*** | 0.004 | ns | ns | ns | ns | ns | ns | b | a | b | ab | - | - | - | - |
| ***sms*** | ns | ns | ns | ns | ns | ns | ns | - | - | - | - | - | - | - | - |
| ***tlr1*** | < 0.001 | ns | ns | ns | ns | ns | ns | a | b | b | b | - | - | - | - |
| ***tlr5*** | < 0.001 | ns | 0.032 | ns | ns | ns | ns | c | b | a | c | - | - | x | y |

**Table S16.** Quantitative expression of *cd4*, *cd8a*, *cd8b*, *igm*, *igt*, *il1b*, *il8*, *il10*, *mhci*, *migd*, *tlr1*, *saa* and *tnfa* in the head kidney of rainbow trout fed dietary treatments for 7 weeks (4 weeks before vaccination and 21 days post-vaccination) and at 4, 24 and 48 hpi. Values are presented as mean ± SD (n = 8 at 21d and n = 9 at 4, 24 and 48 hpi). Different capital letters stand for differences between dietary treatments, different lowercase letters indicate differences among time points, and “x” and “y” denote differences between naïve and vaccinated fish (three-way ANOVA; *p* ≤ 0.05).

| **Parameters** | **Time** | **CTRL** | | **MET** | |
| --- | --- | --- | --- | --- | --- |
|  |  | **Naïve** | **Vac** | **Naïve** | **Vac** |
| ***cd4*** | **21 d** | 1.11 ± 0.45 | 1.06 ± 0.27 | 1.03 ± 0.37 | 1.20 ± 0.61 |
|  | **4 hpi** | 1.05 ± 0.19 | 1.09 ± 0.35 | 0.98 ± 0.31 | 1.05 ± 0.41 |
|  | **24 hpi** | 1.80 ± 0.84 | 1.25 ± 0.38 | 1.25 ± 0.32 | 1.26 ± 0.44 |
|  | **48 hpi** | 1.24 ± 0.58 | 1.09 ± 0.37 | 1.38 ± 0.80 | 1.27 ± 0.61 |
| ***cd8a*** | **21 d** | 1.40 ± 1.14 | 0.72 ± 0.33 | 1.28 ± 0.86 | 0.91 ± 0.45 |
|  | **4 hpi** | 0.92 ± 0.35 | 1.0 ± 0.42 | 1.18 ± 0.72 | 1.20 ± 0.84 |
|  | **24 hpi** | 0.99 ± 0.22 | 1.22 ± 0.57 | 1.07 ± 0.51 | 1.31 ± 0.57 |
|  | **48 hpi** | 1.00 ± 0.44 | 1.07 ± 0.60 | 1.10 ± 0.34 | 1.32 ± 0.89 |
| ***cd8b*** | **21 d** | 1.18 ± 0.72 | 0.55 ± 0.22 | 1.26 ± 0.80 | 0.77 ± 0.60 |
|  | **4 hpi** | 0.72 ± 0.26 | 0.90 ± 0.43 | 0.99 ± 0.75 | 0.98 ± 0.39 |
|  | **24 hpi** | 1.19 ± 0.70 | 1.34 ± 0.69 | 1.07 ± 0.37 | 0.81 ± 0.64 |
|  | **48 hpi** | 0.88 ± 0.52 | 0.94 ± 0.23 | 1.32 ± 0.70 | 1.56 ± 0.81 |
| ***igm*** | **21 d** | 1.04 ± 0.34 | 0.8 ± 0.37 | 0.88 ± 0.43 | 0.78 ± 0.16 |
|  | **4 hpi** | 1.01 ± 0.47 | 0.76 ± 0.43 | 1.06 ± 0.50 | 0.92 ± 0.46 |
|  | **24 hpi** | 1.25 ± 0.68 | 1.14 ± 0.37 | 1.12 ± 0.47 | 1.21 ± 0.64 |
|  | **48 hpi** | 1.32 ± 0.0.32 | 1.26 ± 0.61 | 1.33 ± 0.32 | 1.32 ± 0.40 |
| ***igt*** | **21 d** | 4.63 ± 4.40 | 3.29 ± 2.30 | 5.60 ± 5.38 | 5.59 ± 4.91 |
|  | **4 hpi** | 5.98 ± 2.54 | 4.05 ± 2.71 | 2.72 ± 2.84 | 3.34 ± 2.10 |
|  | **24 hpi** | 6.58 ± 6.48 | 6.68 ± 5.41 | 9.58 ± 5.63 | 8.04 ± 5.47 |
|  | **48 hpi** | 6.28 ± 3.28 | 8.23 ± 5.82 | 6.78 ± 7.23 | 6.85 ± 5.26 |
| ***il1b*** | **21 d** | 1.39 ± 1.19 | 1.65 ± 2.09 | 1.19 ± 0.59 | 1.20 ± 0.82 |
|  | **4 hpi** | 2.97 ± 1.94 | 2.22 ± 1.27 | 2.83 ± 2.41 | 3.47 ± 3.02 |
|  | **24 hpi** | 54.65 ± 86.41 | 2.46 ± 1.30 | 41.29 ± 76.20 | 5.37 ± 9.36 |
|  | **48 hpi** | 3.29 ± 5.48 | 2.84 ± 4.16 | 23.92 ± 67.10 | 1.70 ± 1.41 |
| ***il8*** | **21 d** | 1.00 ± 0.15 | 1.23 ± 0.41 | 0.91 ± 0.22 | 1.13 ± 0.47 |
|  | **4 hpi** | 2.76 ± 1.66 | 1.82 ± 0.80 | 1.60 ± 0.63 | 1.61 ± 0.72 |
|  | **24 hpi** | 5.48 ± 10.34 | 0.97 ± 0.67 | 1.77 ± 0.40 | 1.28 ± 0.70 |
|  | **48 hpi** | 1.60 ± 0.93 | 1.61 ± 0.93 | 1.66 ± 0.44 | 1.44 ± 0.60 |
| ***il10*** | **21 d** | 2.25 ± 3.94 | 1.02 ± 0.66 | 1.74 ± 1.05 | 3.11 ± 4.50 |
|  | **4 hpi** | 1.71 ± 1.50 | 1.79 ± 1.85 | 3.21 ± 4.17 | 3.35 ± 4.60 |
|  | **24 hpi** | 7.43 ± 11.34 | 1.70 ± 1.15 | 4.11 ± 5.80 | 3.58 ± 6.12 |
|  | **48 hpi** | 2.30 ± 2.30 | 2.72 ± 4.38 | 23.08 ± 63.21 | 2.59 ± 2.90 |
| ***mhci*** | **21 d** | 1.65 ± 1.25 | 1.65 ± 1.63 | 1.74 ± 2.07 | 2.03 ± 2.10 |
|  | **4 hpi** | 1.35 ± 0.87 | 1.81 ± 1.91 | 0.87 ± 1.01 | 2.66 ± 2.05 |
|  | **24 hpi** | 3.24 ± 2.32 | 3.19 ± 2.23 | 3.59 ± 3.93 | 2.21 ± 2.38 |
|  | **48 hpi** | 2.81 ± 2.84 | 1.54 ± 2.27 | 3.69 ± 2.99 | 1.77 ± 1.56 |
| ***migd*** | **21 d** | 1.03 ± 0.28 | 1.23 ± 0.32 | 1.08 ± 0.30 | 1.15 ± 0.42 |
|  | **4 hpi** | 1.47 ± 0.46 | 1.36 ± 0.27 | 1.54 ± 0.29 | 1.31 ± 0.45 |
|  | **24 hpi** | 1.88 ± 0.59 | 1.74 ± 0.65 | 2.08 ± 0.76 | 1.78 ± 0.55 |
|  | **48 hpi** | 1.77 ± 0.78 | 1.93 ± 0.70 | 2.01 ± 0.78 | 1.83 ± 0.62 |
| ***tlr1*** | **21 d** | 1.00 ± 0.16 | 1.02 ± 0.15 | 0.92 ± 0.14 | 0.91 ± 0.17 |
|  | **4 hpi** | 1.18 ± 0.15 | 1.15 ± 0.22 | 1.36 ± 0.23 | 1.18 ± 0.29 |
|  | **24 hpi** | 1.46 ± 0.33 | 1.24 ± 0.34 | 1.45 ± 0.32 | 1.33 ± 0.21 |
|  | **48 hpi** | 1.29 ± 0.19 | 1.27 ± 0.23 | 1.37 ± 0.30 | 1.25 ± 0.23 |
| ***saa*** | **21 d** | 3.41 ± 5.87 | 3.97 ± 9.52 | 1.43 ± 1.31 | 2.68 ± 2.96 |
|  | **4 hpi** | 0.43 ± 0.23 | 0.63 ± 0.78 | 8.82 ± 21.97 | 3.04 ± 3.57 |
|  | **24 hpi** | 24.07 ± 45.13 | 3.43 ± 3.24 | 11.71 ± 15.60 | 9.91 ± 11.46 |
|  | 48 hpi | 3.55 ± 2.91 | 3.81 ± 4.02 | 23.68 ± 49.92 | 5.96 ± 11.73 |
| *tnfa* | 21 d | 1.02 ± 0.26 | 1.17 ± 0.37 | 1.03 ± 0.25 | 1.21 ± 0.11 |
|  | 4 hpi | 0.90 ± 0.35 | 1.02 ± 0.31 | 1.05 ± 0.44 | 1.02 ± 0.41 |
|  | 24 hpi | 7.45 ± 9.52 | 1.61 ± 0.38 | 8.30 ± 16.90 | 2.74 ± 3.86 |
|  | 48 hpi | 1.78 ± 0.92 | 1.43 ± 0.54 | 1.26 ± 0.28 | 1.25 ± 0.37 |

|  |  |  |  |  |  |  |  |  | | | |  | |  | | **Time x Diet** | | | | | | | | **Time × Vaccination** | | | | | | | |
| --- | --- | --- | --- | --- | --- | --- | --- | --- | --- | --- | --- | --- | --- | --- | --- | --- | --- | --- | --- | --- | --- | --- | --- | --- | --- | --- | --- | --- | --- | --- | --- |
|  |  |  |  |  |  |  |  | **Time** | | | | **Diet** | | **Vaccination** | | **21d** | | **4 hpi** | | **24 hpi** | | **48 hpi** | | **21d** | | **4 hpi** | | **24 hpi** | | **48 hpi** | |
|  | **Time**  **(T)** | **Diet**  **(D)** | **Vaccination**  **(V)** | **T × D** | **T × V** | **D × V** | **T × D × V** | **21d** | **4 hpi** | **24 hpi** | **48 hpi** | **CTRL** | **MET** | **Nv** | **Vac** | **CTRL** | **MET** | **CTRL** | **MET** | **CTRL** | **MET** | **CTRL** | **MET** | **Nv** | **Vac** | **Nv** | **Vac** | **Nv** | **Vac** | **Nv** | **Vac** |
| ***cd4*** | 0.017 | ns | ns | ns | ns | ns | ns | ab | b | a | ab | - | - | - | - | - | - | - | - | - | - | - | - | - | - | - | - | - | - | - | - |
| ***cd8a*** | ns | ns | ns | ns | ns | ns | ns | - | - | - | - | - | - | - | - | - | - | - | - | - | - | - | - | - | - | - | - | - | - | - | - |
| ***cd8b*** | ns | ns | ns | 0.028 | ns | ns | ns | - | - | - | - | - | - | - | - | - | - | - | - | - | - | B | A | - | - | - | - | - | - | - | - |
| ***igm*** | < 0.001 | ns | ns | ns | ns | ns | ns | c | bc | ab | a | - | - | - | - | - | - | - | - | - | - | - | - | - | - | - | - | - | - | - | - |
| ***igt*** | ns | ns | ns | ns | ns | ns | ns | - | - | - | - | - | - | - | - | - | - | - | - | - | - | - | - | - | - | - | - | - | - | - | - |
| ***il1b*** | < 0.001 | ns | ns | ns | ns | ns | ns | c | ab | a | bc | - | - | - | - | - | - | - | - | - | - | - | - | - | - | - | - | - | - | - | - |
| ***il8*** | 0.003 | ns | 0.009 | ns | 0.002 | ns | ns | - | - | - | - | - | - | - | - | - | - | - | - | - | - | - | - | b | - | a | - | a,x | y | ab | - |
| ***il10*** | ns | ns | ns | ns | ns | ns | ns | - | - | - | - | - | - | - | - | - | - | - | - | - | - | - | - | - | - | - | - | - | - | - | - |
| ***mhci*** | ns | ns | ns | ns | ns | ns | ns | - | - | - | - | - | - | - | - | - | - | - | - | - | - | - | - | - | - | - | - | - | - | - | - |
| ***migd*** | < 0.001 | ns | ns | ns | ns | ns | ns | b | b | a | a | - | - | - | - | - | - | - | - | - | - | - | - | - | - | - | - | - | - | - | - |
| ***tlr1*** | < 0.001 | ns | 0.046 | ns | ns | ns | ns | b | a | a | a | - | - | x | y | - | - | - | - | - | - | - | - | - | - | - | - | - | - | - | - |
| ***saa*** | < 0.001 | 0.030 | ns | ns | ns | ns | ns | b | b | a | a | B | A | - | - | - | - | - | - | - | - | - | - | - | - | - | - | - | - | - | - |
| ***tnfa*** | < 0.001 | ns | ns | ns | 0.010 | ns | ns | - | - | - | - | - | - | - | - | - | - | - | - | - | - | - | - | b | ab | b | b | a,x | a,y | b | ab |
